# RNA-Puzzles Round III: 3D RNA structure prediction of five riboswitches and one ribozyme

**DOI:** 10.1261/rna.060368.116

**Published:** 2017-05

**Authors:** Zhichao Miao, Ryszard W. Adamiak, Maciej Antczak, Robert T. Batey, Alexander J. Becka, Marcin Biesiada, Michał J. Boniecki, Janusz M. Bujnicki, Shi-Jie Chen, Clarence Yu Cheng, Fang-Chieh Chou, Adrian R. Ferré-D'Amaré, Rhiju Das, Wayne K. Dawson, Feng Ding, Nikolay V. Dokholyan, Stanisław Dunin-Horkawicz, Caleb Geniesse, Kalli Kappel, Wipapat Kladwang, Andrey Krokhotin, Grzegorz E. Łach, François Major, Thomas H. Mann, Marcin Magnus, Katarzyna Pachulska-Wieczorek, Dinshaw J. Patel, Joseph A. Piccirilli, Mariusz Popenda, Katarzyna J. Purzycka, Aiming Ren, Greggory M. Rice, John Santalucia, Joanna Sarzynska, Marta Szachniuk, Arpit Tandon, Jeremiah J. Trausch, Siqi Tian, Jian Wang, Kevin M. Weeks, Benfeard Williams, Yi Xiao, Xiaojun Xu, Dong Zhang, Tomasz Zok, Eric Westhof

**Affiliations:** 1 Architecture et Réactivité de l'ARN, Université de Strasbourg, Institut de biologie moléculaire et cellulaire du CNRS, 67000 Strasbourg, France;; 2 Institute of Bioorganic Chemistry, Polish Academy of Sciences, 61-704 Poznan, Poland;; 3 Poznan University of Technology, Institute of Computing Science, 60-965 Poznan, Poland;; 4 Department of Chemistry and Biochemistry, University of Colorado at Boulder, Boulder, Colorado 80309-0596, USA;; 5 Department of Biochemistry, Stanford University School of Medicine, Stanford, California 94305, USA;; 6 Laboratory of Bioinformatics and Protein Engineering, International Institute of Molecular and Cell Biology in Warsaw, 02-109 Warsaw, Poland;; 7 Laboratory of Bioinformatics, Institute of Molecular Biology and Biotechnology, Faculty of Biology, Adam Mickiewicz University, 61-614 Poznan, Poland;; 8 Department of Physics and Astronomy, Department of Biochemistry, and Informatics Institute, University of Missouri-Columbia, Columbia, Missouri 65211, USA;; 9 National Heart, Lung and Blood Institute, Bethesda, Maryland 20892-8012, USA;; 10 Department of Physics and Astronomy, Clemson University, Clemson, South Carolina 29634, USA;; 11 Department of Biochemistry and Biophysics, University of North Carolina at Chapel Hill, Chapel Hill, North Carolina 27599, USA;; 12 Institute for Research in Immunology and Cancer (IRIC), Department of Computer Science and Operations Research, Université de Montréal, Montréal, Québec, H3C 3J7, Canada;; 13 Structural Biology Program, Memorial Sloan-Kettering Cancer Center, New York, New York 10065, USA;; 14 Department of Biochemistry and Molecular Biology, The University of Chicago, Chicago, Illinois 60637, USA;; 15 Department of Chemistry, The University of Chicago, Chicago, Illinois 60637, USA;; 16 Life Sciences Institute, Zhejiang University, Hangzhou 310058, China;; 17 Department of Chemistry, University of North Carolina at Chapel Hill, Chapel Hill, North Carolina 27599-3290, USA;; 18 Department of Chemistry, Wayne State University, Detroit, Michigan 48202, USA;; 19 DNA Software, Ann Arbor, Michigan 48104, USA;; 20 Biomolecular Physics and Modeling Group, School of Physics, Huazhong University of Science and Technology, Wuhan 430074, Hubei, China

**Keywords:** 3D prediction, bioinformatics, force fields, X-ray structures, models, structure quality

## Abstract

RNA-Puzzles is a collective experiment in blind 3D RNA structure prediction. We report here a third round of RNA-Puzzles. Five puzzles, 4, 8, 12, 13, 14, all structures of riboswitch aptamers and puzzle 7, a ribozyme structure, are included in this round of the experiment. The riboswitch structures include biological binding sites for small molecules (*S*-adenosyl methionine, cyclic diadenosine monophosphate, 5-amino 4-imidazole carboxamide riboside 5′-triphosphate, glutamine) and proteins (YbxF), and one set describes large conformational changes between ligand-free and ligand-bound states. The Varkud satellite ribozyme is the most recently solved structure of a known large ribozyme. All puzzles have established biological functions and require structural understanding to appreciate their molecular mechanisms. Through the use of fast-track experimental data, including multidimensional chemical mapping, and accurate prediction of RNA secondary structure, a large portion of the contacts in 3D have been predicted correctly leading to similar topologies for the top ranking predictions. Template-based and homology-derived predictions could predict structures to particularly high accuracies. However, achieving biological insights from de novo prediction of RNA 3D structures still depends on the size and complexity of the RNA. Blind computational predictions of RNA structures already appear to provide useful structural information in many cases. Similar to the previous RNA-Puzzles Round II experiment, the prediction of non-Watson–Crick interactions and the observed high atomic clash scores reveal a notable need for an algorithm of improvement. All prediction models and assessment results are available at http://ahsoka.u-strasbg.fr/rnapuzzles/.

## INTRODUCTION

Our growing knowledge of the biological functions of RNA demands an increased rate of modeling the structures of RNA. Riboswitches are mRNA segments, mostly located in 5′ UTRs that carry out regulatory functions. A riboswitch undergoes conformational changes upon ligand binding and functions as a switch in transcriptional or translational levels. Aptamers are regions of RNA that selectively bind small molecules, whereas riboswitches are natural RNA aptamers embedded in leader sequences of genes. Since riboswitches are functional and may include conformational changes, the 3D structures of riboswitches are of vital importance for understanding the molecular mechanisms of their regulatory functions. One of the aims of computational predictions of 3D RNA structure is to help in the understanding of the binding of small RNA molecules, the conformational changes induced and *in fine* to contribute to the unraveling of the molecular mechanisms of riboswitches.

RNA-Puzzles is a CASP-like (Moult et al. 2016) collective blind experiment for three-dimensional (3D) RNA structure prediction evaluation. The aims are to identify the capacities and bottlenecks in the RNA prediction problem. The structures to be predicted are unknown to the databases and to the modelers and, thus, biases due to prior knowledge are avoided. The prediction methods result from various approaches, but often combine fragment assemblies from known structures present in databases and energy minimizations through various types of force fields adapted to the granularity of the models and the stages of the modeling (for a recent review on the commonly applied algorithms, see Dawson and Bujnicki 2016). Experimental data, newly collected (Cordero et al. 2014), can be also used as constraints during the prediction process. Until now, 16 puzzles have been set up and assessments of six puzzles were previously published (Cruz et al. 2012; Miao et al. 2015). In the recent and ongoing stages of RNA-Puzzles, we have strongly encouraged the development of novel, automatic, and efficient RNA structure prediction algorithms to help the community in understanding real-world RNA structure–function relationships, as well as to promote the development of automated and user-friendly web servers. Since the inauguration of RNA-Puzzles, the field has progressed first and foremost through the numerous discussions and exchanges between the various modeling groups. This has led to agreed protocols for delivery of models, descriptions of computations, and assessments. At the same time, the automatization of the modeling processes has steadily progressed. At this stage, it is probably still too early to offer a comparative analysis of the prediction and modeling methods.

Here we report a third round of RNA-Puzzles and we focus on the prediction of RNA riboswitches and ribozymes, evaluated on the basis of six RNA structures: the SAM-I riboswitch aptamer, the SAM-I/IV riboswitch, the *ydaO* riboswitch, the ZTP riboswitch, the L-glutamine riboswitch, and the Varkud satellite ribozyme. These molecules are functionally significant as they can bind ligands, may include conformational changes, or can catalyze chemical reactions. Contributing to the stringency of this round, all six molecules included regions without homology to previously solved structures, and in most cases the problem required modeling the entire structure de novo. According to the prediction results, we discuss several critical aspects of RNA 3D structure predictions: (i) the prediction of RNA noncanonical contacts, (ii) the prediction of structural topology, and (iii) the understanding of small molecule binding and the induced conformational changes.

We find that RNA 3D structure prediction has already achieved a high level of accuracy for template-based and homology-based structure predictions and, thus, can already contribute significantly to our understanding of the underlying molecular mechanisms in some cases. The prediction of ligand binding and the resulting conformational changes are also possible but cannot be guaranteed. For a large de novo structure, the prediction is still a difficult endeavor.

## RESULTS AND DISCUSSION

### The five RNA-Puzzles on riboswitches

#### Puzzle 4: SAM-I riboswitch aptamer

This SAM-I riboswitch problem is an aptamer where the P3 helix is engineered as an extended helix (Baird et al. 2012). It binds an *S-*adenosyl methionine (SAM) molecule in its center and can bind L7Ae-like proteins (YbxF and YlxQ) at the K-turn module. The 126-nucleotide (nt)-long sequence is the following:

5′-GGCUUAUCAAGAGAGGUGGAGGGACUGGCCCGAUGAAACCCGGCAACCACUAGUCUAGCGUCAGCUUCGGCUGACGCUAGGCUAGUGGUGCCAAUUCCUGCAGCGGAAACGUUGAAAGAUGAGCCA-3′

After the prediction deadline, the 2.8 Å diffraction resolution structure was deposited in PDB with ID 3V7E. Before the experiment, homologous structures identical to this particular riboswitch were already available (Stoddard et al. 2010). The SAM binding position is identical to the prior structure (see Supplemental Fig. S1), and the ligand-free state (3IQP) also adopts the same topology. The L7Ae/YbxF protein-binding region is easily detected as a K-turn module. The RMSD of aligned parts between previously available 3IQR and the new protein-bound coordinates 3V7E is <1 Å. The difference lies on the engineered P3 helix and the minor deviations of the P4 helix. Therefore, this puzzle is a template-based prediction of high-resolution modeling.

#### Puzzle 8: SAM-I/IV riboswitch

The SAM-I/IV riboswitch aptamer is a structure with homologies to both SAM-I and SAM-IV families (Trausch et al. 2014). It binds SAM in a region similar to the SAM-I riboswitch but may originate from a different ancestor. The sequence is 96-nt long:

5′-GGAUCACGAGGGGGAGACCCCGGCAACCUGGGACGGACACCCAAGGUGCUCACACCGGAGACGGUGGAUCCGGCCCGAGAGGGCAACGAAGUCCGU-3′

The structure was solved to 2.95 Å with PDB ID 4L81. Before this crystal structure of the SAM-I/IV riboswitch aptamer was published, the SAM-I structure was known and available during RNA-Puzzle modeling. Nevertheless, the SAM-I/IV riboswitch aptamer exhibits distinct peripheral tertiary domains and pseudoknots. The prediction of SAM-I/IV is an appropriate application of RNA structure prediction programs for understanding biological problems with unknown RNA structures. The known clues could be explored, such as the structural topologies of SAM-I and SAM-IV, to predict parts of the unknown structure, while other parts required de novo modeling.

#### Puzzle 12: *ydaO riboswitch*

Two cylclic diadenosine monophosphate (c-di-AMP) molecules bind the *ydaO* riboswitch, which is involved in sporulation, osmotic stress responses, and cell wall metabolism, in two pseudo-symmetry-related pockets (Ren and Patel 2014). The sequence of the 108-nt *ydaO* riboswitch is as follows:

5′-AUCGCUGAACGCGGGGGACCCAGGGGGCGAAUCUCUUCCGAAAGGAAGAGUAGGGUUACUCCUUCGACCCGAGCCCGUCAGCUAACCUCGCAAGCGUCCGAAGGAGAA-3′

The structures of the complex were solved at resolutions 2.65 Å (binding with c-di-dAMP, PDB 4QLN) and 2.72 Å (binding with c-di-AMP, PDB 4QLM). Although a c-di-GMP bound riboswitch was previously solved, the *ydaO* riboswitch is a new structure topology and is difficult to predict. When predicting the two c-di-AMP binding pockets, non-Watson–Crick edges of specific aptamer nucleotides directly contacting the ligands were unknown.

#### Puzzle 13: ZTP riboswitch

A ZTP (5-amino 4-imidazole carboxamide riboside 5′-triphosphate) riboswitch can up-regulate de novo purine synthesis in response to increased intracellular levels of ZTP or ZMP (Trausch et al. 2015). The sequence of the ZTP riboswitch structure is of 60 nt:

5′-GGGUCGUGACUGGCGAACAGGUGGGAAACCACCGGGGAGCGACCCGCCGCCCGCCUGGGC-3′

Two PDB structures were solved at resolutions 2.5 Å (PDB 4XW7) and 1.8 Å (PDB 4XWF), respectively. Despite the lack of a homologous structure, the secondary structure of the riboswitch is relatively simple and the size of the structure is small, which facilitated the prediction.

#### Puzzle 14: L-glutamine riboswitch

The L-glutamine riboswitch goes through dramatic conformational changes in the P3 helix upon glutamine binding (Ren et al. 2015). The length of the structure is 61 nt. Two sequences, corresponding to constructs used to crystallize ligand-bound and ligand-free versions of the aptamer, were as follows:

5′-CGUUGACCCAGGAAACUGGGCGGAAGUAAGGCCCAUUGCACUCCGGGCCUGAAGCAACGCG-3′ (Bound)
5′-CGUUGGCCCAGGAAACUGGGUGGAAGUAAGGCCCAUUGCACUCCGGGCCUG AAGCAACGCU-3′ (Free)

The ligand-free state structure was solved at resolution 3.1 Å and deposited in PDB with ID 5DDO, while three L-glutamine bound structures were solved: 5DDP at 2.3 Å, 5DDQ at 2.4 Å, and 5DDR at 2.61 Å resolution. Although 5DDQ and 5DDR were solved in Mn^2+^-soaked and Cs^+^-soaked conditions, their structural differences from 5DDP are subtle. The structural modules GAAA tetra-loop and U1A-protein-binding loop, engineered to replace Loops L2 and L3, were interesting in prediction. For conformational changes, the nucleotides G22 and G23, disordered in the free state, form critical long-range interactions in the ligand-bound state. The correct prediction of these interactions was expected to influence overall prediction accuracy.

### The RNA-Puzzle on a ribozyme

#### Puzzle 7: the Varkud satellite (VS) ribozyme

Besides riboswitches, we also report in this round of experiments the prediction of a self-cleaving ribozyme. The Varkud satellite ribozyme (Suslov et al. 2015), as part of VS RNA, is the largest known small nucleolytic ribozyme. The 185-nt sequence covers residues 601–785 of VS RNA:

5′-GCGCUGUGUCGCAAUCUGCGAAGGGCGUCGUCGGCCCGAGCGGUAGUAAGCAGGGAACUCACCUCCAAUGAAACACAUUGUCGUAGCAGUUGACUACUGUUAUGUGAUUGGUAGAGGCUAAGUGACGGUAUUGGCGUAAGCCAAUACCGCGGCACAGCACAAGCCCGCUUGCGAGAUUACAGCGC-3′

Two single site mutant structures of the VS ribozyme were solved to the same resolution of 3.07 Å, PDB 4R4P and 4R4V. The structure deviations between the two structures are slight. However, the structure is large and no homologous structure was available before the experiment, making it a difficult problem. Furthermore, the RNA crystallized as a dimer, and modelers were challenged with predicting a complex with a total size of 370 nt, the largest RNA-Puzzle problem to date.

### Experimental data description

The Das group provided “fast-track” experimental data to all the modelers for puzzles 7, 8, 12, 13, and 14. One-dimensional chemical mapping using SHAPE, CMCT, DMS, and hydroxyl radical footprinting and multidimensional chemical mapping measurements based on mutate-and-map (M^2^) and multiplexed •OH cleavage analysis (MOHCA-seq, for targets 12–14) were acquired as described in Cordero et al. (2014), Kladwang et al. (2014), and Cheng et al. (2015a). Data were distributed via the private RNA-Puzzles website or via entries with anonymized sequences in the RNA Mapping Database (Cordero et al. 2012); deposition IDs are summarized in Supplemental Table S8.

### Overall comparison results

#### Assessment methods

The automatic structure comparison workflow for assessment was set up as reported in previous rounds of RNA-Puzzles (Cruz et al. 2012; Miao et al. 2015). In this round of experiments, four out of the six puzzles were solved with more than one crystal structure. When multiple structures were available, we assumed the RNA might populate a diverse ensemble of structures and every structure is a possible native structure. Hence, when assessing the prediction quality of a structure, comparisons were made with all available crystal structures. Up to now, there is no single or universal metric that can be considered as the major determinant of the overall accuracy of a predicted model. Therefore, we use a set of metrics to assess all the models. Because it is the most commonly used and obvious metric, the root mean square deviation (RMSD) between the predicted models and the crystal structure is used for ranking the models. RMSD is a metric for global topology comparisons, but it spreads the errors all over the structure. Indeed, we can find some special cases where the RMSD shows a different ranking from other metrics. Different metrics were used to assess different aspects of the predictions: RMSD stands for the global similarity of all the atoms; deformation index (DI) and the complete deformation profile matrix (DP) stand for prediction accuracy of the nucleotide interactions, while the interaction network fidelity (INF) assesses the interaction accuracies at different levels (Parisien et al. 2009); the Clash score evaluated by MolProbity (Chen et al. 2010) assesses the atomic harmony of the structure, and the mean of circular quantities (MCQ) score (Zok et al. 2014) assesses the structural similarity with the native structure in the torsion angle space. Each of those metrics, because they assess very different structural characteristics, has advantages and drawbacks. Thus, DI, which stands for the local pairwise superimpositions, does not show the differences when all predicted structures are far away from the native structure. INF defines the quality of a certain type of predicted interaction but not all the elements. Clash score only demonstrates the reasonability of the atomic distances and is not discriminative. MCQ compares the dihedral angles without considering bond lengths or bond angles. We now add radar diagrams (Supplemental Figs. S21–S27) to give a general idea and an overview of the scores related to the first ranked and best RMSD models of the participating groups. The advantage of using a set of metrics assessing various molecular characteristics is that it shows the qualities and deficiencies of the various algorithms as a function of the size and type of RNA molecule being predicted.

### The five RNA-Puzzles on riboswitches

#### Puzzle 4: the SAM-I riboswitch aptamer (see Fig. 1; Supplemental Figs. S1, S2)

**FIGURE 1.**
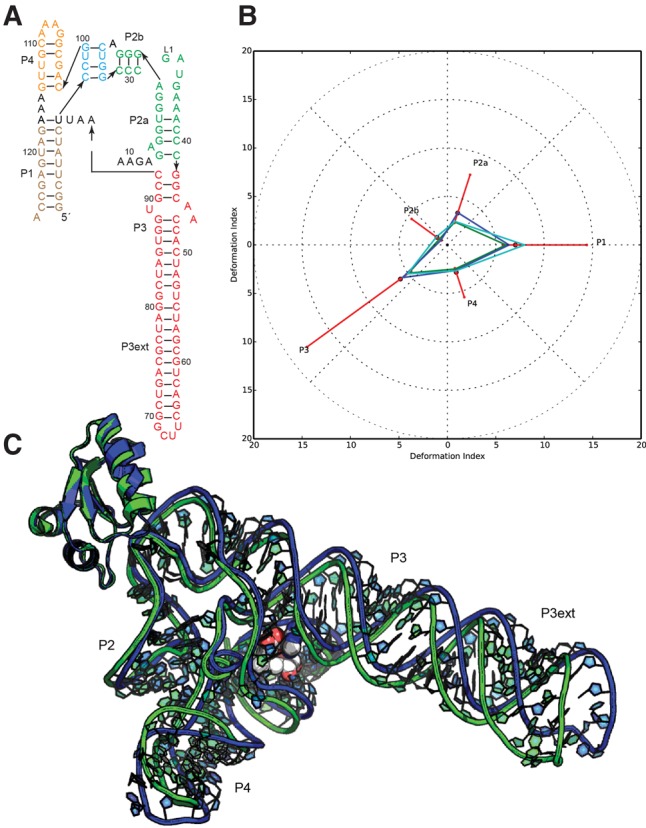
Puzzle 4: the SAM-I riboswitch aptamer. (*A*) Secondary structure and (*B*) module-based deformation profile values for the three predicted models from different groups with lowest RMSD: Chen model 2 (green), Bujnicki model 1 (blue), and Santalucia model 1 (cyan). (Radial red lines) The minimum, maximum, and mean DP values for each domain. (*C*) Structure superimposition between native structure (green) and best predicted model (blue, Chen model 2).

Because of the availability of homologous templates, such as 3IQP, the prediction accuracy of Puzzle 4 is extremely high. As shown in Supplemental Table S1, 28 out of 30 total predicted structures have a RMSD within 6 Å, while 10 prediction models from the Chen laboratory are within 3.5 Å. The Watson–Crick base pairs were perfectly predicted. Generally, a better prediction always includes a better identification of non-Watson–Crick pairs and of stacking contacts. Further, we find the prediction models from the Chen laboratory are well optimized for atomic clashes. This indicates that a very good level of high-resolution homology modeling of RNA structures has been achieved (see Fig. 1). The Das group provided predictions of SAM binding and the orientation of the SAM that are very close to the native binding position, shown in Supplemental Figure S3. In homology modeling, the SAM binding region can also be inferred from known templates. The contacts between SAM and the riboswitch are compared in Supplemental Figure S4; most of the contacts (mainly hydrogen bonds) predicted by Das model 1 have been predicted in a correct manner. Several groups also predicted the fold of the YbxF protein and its binding of the SAM I K-turn. The availability of prior templates for K-turns bound to proteins such as L7Ae allowed these groups to achieve near-atomic accuracy (Fig. 1). Most of the groups predicted the protein at the right positions, since the L7Ae-Kturn binding is well known.

#### Puzzle 8: the SAM-I/IV riboswitch (see Fig. 2; Supplemental Figs. S5, S6)

**FIGURE 2.**
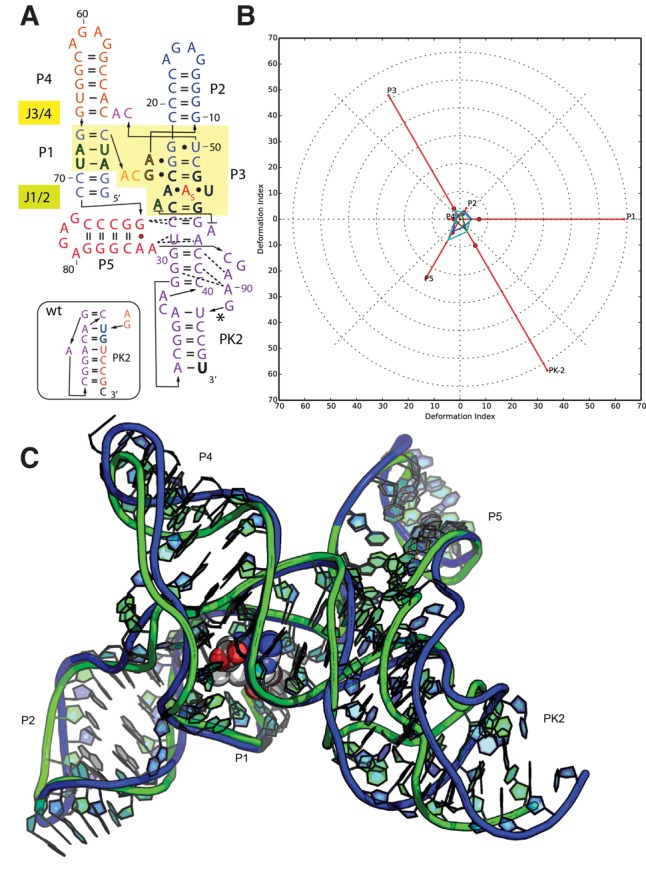
Puzzle 8: SAM-I/IV riboswitch. (*A*) Secondary structure and (*B*) module-based deformation profile values for the three predicted models from different groups with lowest RMSD: Das model 3 (green), Bujnicki model 7 (blue), and Chen model 3 (cyan). (Radial red lines) The minimum, maximum, and mean DP values for each domain. (*C*) Structure superimposition between native structure (green) and best predicted model (blue, Das model 3).

Although no high-resolution structural template exists for this puzzle, structural clues about the SAM-I/IV riboswitch can be derived from SAM-I riboswitch structures. Potentially, similar distant-homology-based predictions could be an important application of RNA structure prediction in interpreting molecular mechanism and biological functions. Even if only four out of 42 predictions were predicted below 7 Å RMSD, the best one is still within 5 Å RMSD (Supplemental Table S2). This model, from the Das group, correctly predicts helix P5 stacked against the backbone of pseudoknot PK2 in an unexpected manner (Fig. 2). Even at 11 Å RMSD, many prediction models could potentially be helpful in understanding the structure. In the top ranking cases, Watson–Crick pairs and stacking were predicted to a very high level, but the prediction of the non-Watson–Crick pairs still needs to be improved. Although no SAM binding was predicted in this puzzle, the SAM binding site can be inferred from related structures since they maintain identical contacts. A comparison between SAM-I riboswitch and SAM-I/IV is shown in Supplemental Figure S6.

#### Puzzle 12: the ydaO riboswitch (see Fig. 3; Supplemental Figs. S7, S8)

**FIGURE 3.**
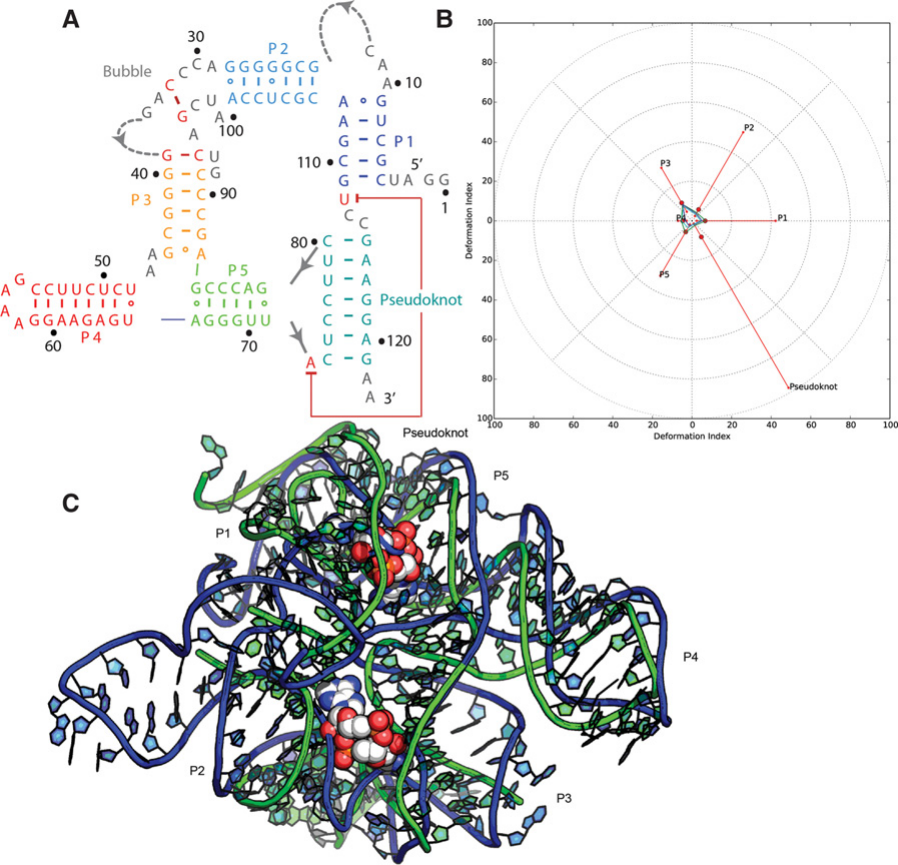
Puzzle 12: *ydaO* riboswitch. (*A*) Secondary structure and (*B*) module-based deformation profile values for the three predicted models from different groups with lowest RMSD: Ding model 12 (green), Bujnicki model 8 (blue), and Das model 7 (cyan). (Radial red lines) The minimum, maximum, and mean DP values for each domain. (*C*) Structure superimposition between native structure (green) and best predicted model (blue, Ding model 12).

The *ydaO* riboswitch can be defined as a “difficult case” because of its relatively large size, its lack of homology to any previously solved RNA structures, and because of binding effects of the two ligands, which requires special consideration of the availabilities of the non-Watson–Crick edges. The RMSD of the 51 submitted predictions range from 10 to 36 Å with an average value of 16.6 Å, as shown in Supplemental Table S3. The P4 and P5 helical regions, longer and thus more stable, are better predicted, while P2 and P3 are worse. The bubble between P2 and P3 helix was mostly unresolved in the X-ray map, implying that this region could be less stable in structure. Nevertheless, independent crystallographic solutions of homologous c-di-AMP structures (Gao and Serganov 2014; Jones and Ferré-D'Amaré 2014) (also released after RNA-Puzzle 12 modeling) showed strong agreement with all resolved parts of the crystal structure considered herein, suggesting that the overall fold is well defined and a valid target for prediction. Many of the predicted structures do not fully consider the binding of the two c-di-AMP ligands, but the global topologies of the top ranking models are still visually similar to the X-ray structure (Ding group model in Fig. 3). The pseudoknot and the bubble are difficult to predict in this puzzle, and the superimposition of the best model is shown in Supplemental Figure S8. The pseudoknot is very well predicted while the bubble region is poor in all models, likely due to an incorrect secondary structure used by most modelers. This may be largely related to the flexibility of the structure, as the pseudoknot includes many base pair interactions and is stable. However, the bubble is too flexible so can only be partly solved in crystal and the prediction is thus more difficult.

#### Puzzle 13: the ZTP riboswitch (see Fig. 4; Supplemental Figs. S9, S10)

**FIGURE 4.**
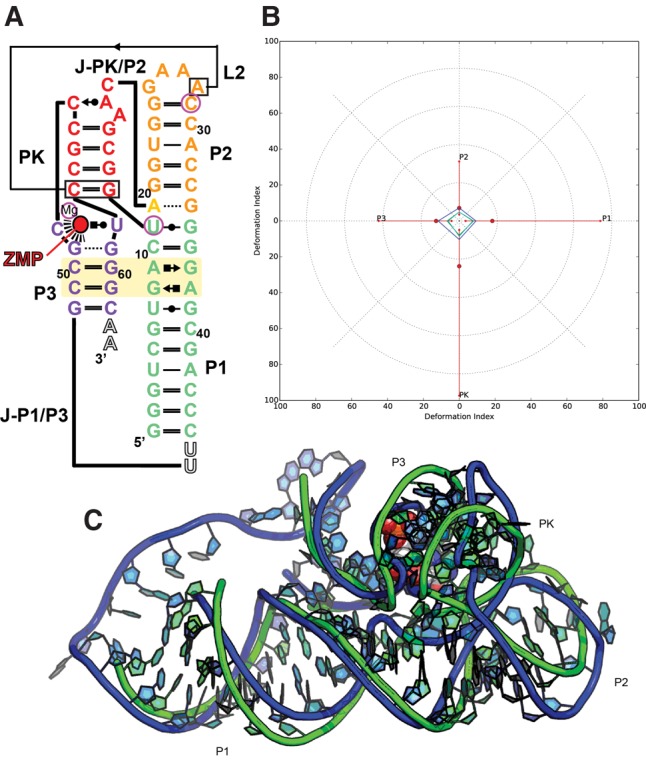
Puzzle 13: ZTP riboswitch. (*A*) Secondary structure and (*B*) module-based deformation profile values for the three predicted models from different groups with lowest RMSD: Das model 7 (green), Chen model 5 (blue), and Bujnicki model 3 (cyan). (Radial red lines) The minimum, maximum, and mean DP values for each domain. (*C*) Structure superimposition between native structure (green) and best predicted model (blue, Das model 7). The bound ZMP is shown in red.

Like Puzzle12, the ZTP riboswitch is a full de novo prediction, but the size of the structure is relatively small and mainly comprised of Watson–Crick interactions. Thus, the top two predictions, from the Das group, achieved RMSD within 6 Å, strikingly similar to the native topology, shown in Supplemental Table S4. According to Figure 4, we find that the predicted structure adopts exactly the same fold as the native structure, except that the curvature of the helix deviates slightly. The predicted structure gives a structure model of the single-stranded loop between P1 helix and P3 helix, which is not solved by crystallography. This loop region may be too dynamic for a unique conformation and the predicted structure is also disordered. For the ZMP binding, the coordinates given by the Das laboratory model 7 were quite close to the native binding region, but with an opposite orientation that is less buried in the RNA structure. As shown in Supplemental Figure S10, the native ZMP binding position is between Das model 1 and model 7.

#### Puzzle 14: the L-glutamine riboswitch (see Fig. 5; Supplemental Figs. S11–S14)

**FIGURE 5.**
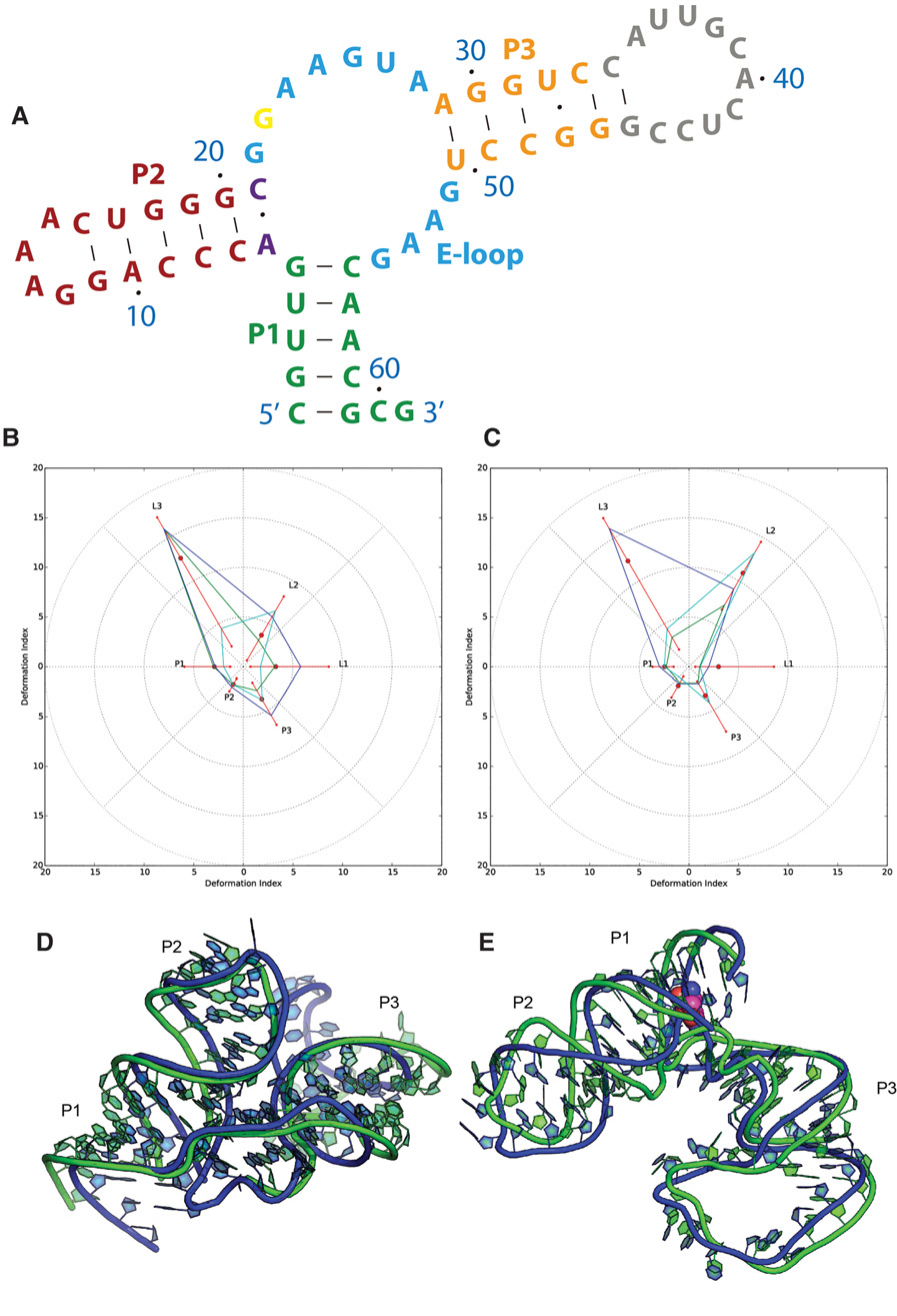
Puzzle 14: L-glutamine riboswitch. (*A*) Secondary structure and (*B*) module-based deformation profile values, measured from free state structure, for the three predicted models from different groups with lowest RMSD: Das post-experiment model 2 (green), Ding post-experiment model 8 (blue), and Chen post-experiment model 2 (cyan). (Radial red lines) The minimum, maximum, and mean DP values for each domain. (*C*) Module-based deformation profile values, measured from bound state structure, for the three predicted models from different groups with lowest RMSD: Bujnicki pre-experiment model 2 (green), Ding post-experiment model 8 (blue), and Chen post-experiment model 2 (cyan). (*D*) Free state structure superimposition between native structure (green) and best predicted model (blue, Das post-experiment model 2). (*E*) Bound state structure superimposition between native structure (green) and best predicted model (blue, Bujnicki pre-experiment model 2).

The L-glutamine riboswitch is the first RNA molecule in RNA-Puzzles for which a large conformational change upon ligand binding has been experimentally captured. The sequences of molecules used to crystallize both the free and bound states were released for the prediction experiment. This puzzle points to the important question of how well RNA conformational change can be predicted by state-of-the-art methods. If a prediction can achieve reasonable quality in both states, it increases the likelihood that de novo RNA structure prediction might provide useful hypotheses of explanations for molecular mechanism in the near future. Structure comparisons between predictions and native structures are available in Figure 5, while 2D heat maps of deformation profiles are demonstrated in Supplemental Figures S11–S13.

Further, to begin quantifying the help of the inclusion of additional experimental data, Puzzle 14 was divided into “pre-experiment prediction” and “post-experiment prediction.” Fifty-one predicted structures of free state and 64 of bound state are listed in Supplemental Table S5 and Supplemental Table S6. The best prediction of free state is from the “post-experiment” model 2 of the Das laboratory that is 6.5 Å RMSD away from native structure, while the best for bound state is from “pre-experiment” model 2 of the Bujnicki laboratory, which is 5.0 Å RMSD away from the native structure. Interestingly, these most accurate predictions were made with Rosetta FARFAR methods and are capable of explaining the ligand-induced conformational change, but the worst cases in prediction are quite far away, ∼20 Å RMSD. In the free state prediction (Supplemental Table S5), only two of the top 10 models are pre-experiment ones: Das pre-experiment models 1 and 9 ranking at second and eighth. However, six out of the top 10 models are pre-experiment predictions in the bound state prediction, while the top three models are also pre-experiment ones. Consequently, utility of experimental data (or at least the additional time allowed for modeling) on the free state prediction appears clear; but such improvement is not detected in the bound state modeling. The Bujnicki pre-experiment models 1–4 are the best models for bound state predictions, but after the integration of experimental data, these models were not recognized as the best ones and were not included in post-experiment models, suggesting that either the experimental data were misleading or were not helpful in late-stage refinement of these models.

A Loop E motif, present in the Puzzle14 structure, is a critical structural module in RNA structure and was recognized previously based on sequence conservation (Ames and Breaker 2011). The prediction of the Loop E structure is compared in Supplemental Figure S14 for both free and bound states. In the free state, only Das group post-experiment model 2 gave the right prediction. In the bound state, Bujnicki group pre-experiment model 2 and Das group pre-experiment model 6 are the best predicted ones. The well-predicted loop E modules are always detected in the top ranking predictions, which underscores the importance of predicting correctly any well-known structural modules for the final resulting model.

### The RNA-Puzzle on a ribozyme

#### Puzzle 7: the Varkud satellite ribozyme (see Fig. 6; Supplemental Figs. S15–S13)

**FIGURE 6.**
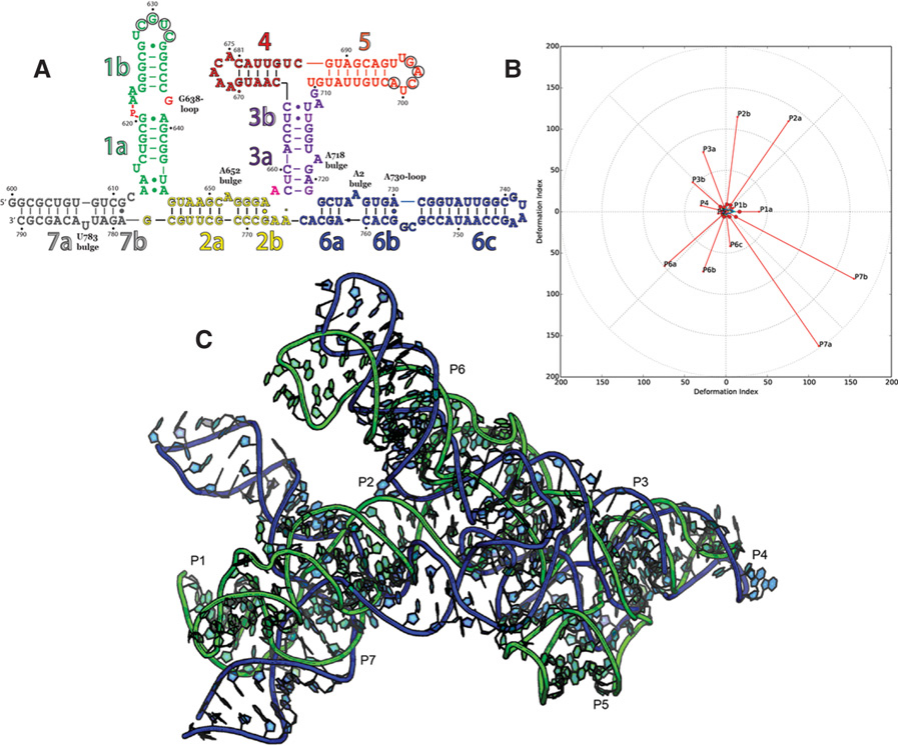
Puzzle 7: Varkud satellite ribozyme. (*A*) Secondary structure and (*B*) module-based deformation profile values for the three predicted models from different groups with lowest RMSD: Das model 1 (green), Chen model 5 (blue), and Bujnicki model 5 (cyan). (Radial red lines) The minimum, maximum, and mean DP values for each domain. (*C*) Structure superimposition between native structure (green) and best predicted model (blue, Das model 1).

As the only ribozyme in this round of RNA-Puzzles and the largest of the small nucleolytic ribozymes, the VS ribozyme was very difficult to predict de novo, although much structural data and many models were previously available (Wilson and Lilley 2011). The RMSD of the predictions range from 20 to 60 Å, with a mean value of 29 Å. Figure 6B demonstrates that the best models from, e.g., the Das group model 1, accurately predict the important modules. Nevertheless, the relative orientation between the different helical structures in 3D space is difficult to predict. In Supplemental Figure S16, all of the 7 hairpins and internal stems (P1–P7) are compared between native and prediction. Although the predictions are each similar to the native, small deviations of non-Watson–Crick interactions then led to global topological differences clearly detected in Supplemental Figure S16A,D,F. In particular, the P2–P3–P6 junction was incorrectly assumed in prior modeling efforts and RNA-puzzle modeling herein. Using “standard” rules (Lescoute and Westhof 2006) and characterization of the junction in isolation (Lafontaine et al. 2001), P3 and P6 were expected to be coaxially stacked as there are no unpaired nucleotides between them; but the crystal structure revealed these helices to be separated.

The native structure adopts a more expanded state than the predicted structures. Still, we find that the prediction of non-Watson–Crick interactions is poor, as shown in Supplemental Table S7, although the structure topology could be softened by these noncanonical interactions. However, such deviations in local structure may result in topological change between different structural domains. As a further challenge, the RNA crystallized as a dimer, swapping the stem P1 holding the cleavage site between the two partners. These reasons explain the poor global RMSD that could be achieved for this structure. Therefore, further efforts are still needed in predicting large multimeric structures de novo.

### General comments

#### Model ranking

For each Puzzle, predictors were allowed to submit up to 10 structural models that they ranked from the most reliable model down to the least reliable ones. This ranking should constitute a direct measure of the quality of the overall scoring function and especially of its correlation with structural models as derived from crystallography. As for automated structure prediction web servers, one is likely to consider only the first few models. However important, ranking the prediction models is a nontrivial but practical step.

If we only take the first model into consideration, the accuracies of the prediction worsen substantially. For example, for Puzzle 12, a model of the Ding group gives the best RMSD to the X-ray structure, but that model was ranked worst (12th of 12 submissions) from that group during blind modeling. The same group's top-ranked model is only the 18th best model and brings a 5 Å decrease in RMSD compared with their most accurate model, as assessed post facto. The bound state of Puzzle 14 is another case where models with accurate global folds were submitted but were ranked low by the modeling groups on their ranked lists. To give a better view on relationships between highly scored models and their ranking with respect to different metrics, we provide radar diagrams illustrating the relative rankings built for the first models and for the best RMSD models (Supplemental Figs. S21–S27). They clearly show that the best RMSD model is not necessarily the best in terms of all measures. In extreme cases, highly scored models in one ranking can be significantly worse than top-ranked models in the other ranking. Thus, we should be aware of how important it is to consider various evaluation measures when assessing the results of 3D structure prediction.

### Crystal derived B-factor values and the quality of the predictions

In Supplemental Figures S2, S5, S7, S9, S11–S13, S15, the 2D heat maps of the deformation profiles are used to demonstrate the prediction accuracy locally in each part between the predicted and the crystal RNA structures. The majority of the heat maps point to high accuracies of the top ranking models, except Puzzle 12 (RMSD 10 Å) and Puzzle 7 (RMSD 20 Å). According to Supplemental Tables S1–S7, there is a good correlation between RMSD and deformation profile (DI all). Generally, accurate prediction requires good relative positions between local parts. Further, the B-factors plotted are aligned on the left of the heat maps as histogram plots. We find many of the badly predicted regions correspond to higher B-factor values, which suggests that these nucleotides or large RNA segments are highly mobile or that several of the nucleotide atoms may not have clear density in the electron density map. In such cases, the X-ray data may not be sufficient to fully determine the coordinates of such nucleotides and the derived coordinates may present large errors. For example, the high B-factor region of Puzzle 4 (three residues in the apical loop of the P3 extension) corresponds to those parts with the highest deformation profile and thus potentially badly modeled, either by the crystallographers or the modelers, or that this region does not assume a single conformation, especially in solution. Examination of the electron densities of these three high B factor residues in Supplemental Figure S17 reveals that the coordinates of the crystal structure do not fit the electron densities well. Hence, it is difficult to assess the accuracy of the predictions for those nucleotides. Leaving out such residues, the majority of the local structure parts have been predicted quite accurately. This suggests that RNA 3D structure prediction may help build better structural models in crystallography or in conjunction with other structural determination methods.

## MATERIALS AND METHODS

In the following, we briefly introduce the methods used and focus on the new updates and special treatments in the prediction.

### Adamiak group

In all the puzzles, a fully automated prediction method (Popenda et al. 2012) provided by RNAComposer (http://rnacomposer.ibch.poznan.pl and http://rnacomposer.cs.put.poznan.pl) was applied to conduct the RNA 3D structure prediction. RNAComposer is a knowledge-based method that employs automated fragment assembly, based on the secondary structure tree graph representation and homology of structural elements. At present the RNAComposer dictionary contains as much as 490,000 3D structure elements. Models delivered by RNAComposer are energy minimized and refined.

The prediction fidelity of RNAComposer depends critically on the accuracy of the RNA secondary structure, used as an input (Popenda et al. 2012; Purzycka et al. 2015). Therefore, secondary structures predicted in silico using tools integrated in the RNAComposer system were adjusted according to experimental data, if available. The Adamiak group also applied Rfam (Gardner et al. 2009) to identify conserved base pairs (Puzzle 13). Information about pseudoknots was obtained by manual analysis of the experimental data (Puzzles 7, 8, and 13) and/or based on the literature review (Puzzles 12 and 13). Supplemental Table S9 presents input RNA secondary structure topologies applied to the considered puzzles. Information about pairing patterns within the pseudoknots was introduced into the RNAComposer dot-bracket annotated input using square brackets. The structure of each potential pseudoknot was additionally refined using distance restraints derived from canonical A-RNA structure (Supplemental Table S9) applying the described procedure (Huang et al. 2013). In some cases, the generated 3D structures did not correlate with long-distance interactions or relative positioning of the helixes deduced from the provided experimental data (i.e., Puzzle 8). This prompted us to develop new functionality of the RNAComposer system (Antczak et al. 2016) that allows the user to introduce a particular, user-defined 3D element for the structure assembly. This functionality was subsequently utilized in solving Puzzles 13 and 14. Difficulties in predicting some interactions from the experimental data resulted in another improvement of the RNAComposer system. New algorithms are being developed that will increase the pool of promising 3D elements that can be applied for 3D structure assembly by allowing the user to explore RNA FRABASE (Popenda et al. 2008, 2012) using new wild card characters. Such characters will be introduced in the definition of the query patterns.

All 3D models were evaluated using the criteria of total energy and right geometry to exclude knotted structures, and those that did not fulfilled experimental restraints criterion. Final 3D models were verified using RNApdbee server (Antczak et al. 2014), available at http://rnapdbee.cs.put.poznan.pl, to confirm that input secondary structure topology is preserved. Our RNA 3D models are highly ranked according to measured INF_WC_.

The Adamiak group was able to generate hundreds of 3D structure models very fast. The major limitation was a manual analysis of provided experimental data, and identification of these 3D models that agreed best with the experimental data within the given time.

#### Puzzle 4

The Adamiak group focused on the prediction of the RNA–protein complex (Fig. 1). The high level of homology for *B. subtilis* YbxF protein (sequence: GSYDKVSQAKSIIIGTKQTVKALKRGSVKEVVVAKDADPILTSSVVSLAEDQGISVSMVESMKKLGKACGIEVGAAAVAIIL) allowed us to obtain a protein 3D model based on homologous modeling with I-TASSER (Yang et al. 2015).

RNA secondary structure was extracted from the SAM-I riboswitch of *B. subtilis* (PDB id: 3NPB) and used to predict RNA 3D structure in the fully automated mode of RNAComposer. Three-dimensional models of RNA/protein complexes were obtained using HADDOCK (Dominguez et al. 2003). The Adamiak group defined protein–RNA interfaces based on the structures of L7Ae bound to the K-turn motif in box C/D RNA (1RLG—*A. fulgidus* L7Ae, 1SDS—*M. jannaschii* L7Ae, 3PLA—*S. solfataricus* L7Ae) as well as flexible regions (“semiflex” and “fullyflex”) in RNA and protein. From obtained 3D models of RNA/protein complexes, five were chosen that showed the highest score returned by HADDOCK and the number of well-defined, specific hydrogen bonds between RNA and protein. For model 3, the RMSD value (4.008 Å) of the predicted complex was nearly identical to the RNA component itself (RMSD 4.091 Å). Outermost results were obtained when the “fullyflex” option was used upon docking.

#### Puzzle 8

After introduction of additional restraints on the residues constituting the pseudoknot (Supplemental Table S9), obtained 3D models were inspected for the agreement with the provided Mutate-and-Map data. However, the predicted 3D models did not preserve the tertiary interactions deduced from the provided experimental data. Therefore, the Adamiak group decided to submit the best two 3D structure models based on the total energy calculated by RNAComposer. Those models displayed very good INF_NWC_ (0.612 and 0.548) as depicted on Supplemental Figure S23.

#### Puzzle 12

The RNA secondary structure topology was based on published data (Nelson et al. 2013). Topologies with 5 or 6 base pairs (bp) forming the pseudoknot were used as the RNAComposer input (Supplemental Table S9). Additional base pairs 27–101 and 32–37 were also introduced based on expert analysis of initially predicted 3D models.

#### Puzzle 13

Secondary structure was developed based on the data published by Kim et al. (2015). New functionality of RNAComposer was implemented (Antczak et al. 2016), and user-defined 3D structure elements for the internal loop (7-UGACUGGCGAACAG-20; 33-CGGGGAG-39 and the single strand: 7-UGACUGGCGAACAG-20) were introduced. Those structural elements were identified in RNA FRABASE (Popenda et al. 2008, 2010) using our own scripts. Thirty-three structurally diverse 3D elements displaying 25%–42% of sequence identity and nine with the best sequence identity were chosen for the internal loop and for the single strand element, respectively. The Adamiak group applied all combinations of those 3D elements in the automated model assembly by RNAComposer and obtained 297 highly different resultant 3D structures. Subsequently, the final 3D models were chosen according to highest agreement with the experimental data and acceptable total energy delivered by RNAComposer. The 3D structure model displaying the best compatibility with experimental data was submitted. This model shows the best INF_WC_ score out of all (55) ranked 3D structures (Supplemental Fig. S25).

#### Puzzle 14

RNA secondary structure generated using RNAfold (Zuker and Stiegler 1981) was analyzed and adjusted. The G5–C55 base pair was substituted with G23–C55. This change allows RNAComposer to automatically recognize the internal loop: 23-GAAGUAA-29; 50-UGAAGC-55, as E-loop motif. Such a motif was previously reported also for glutamine aptamers (Ames and Breaker 2011). RNAComposer generated ten 3D structures. Two structures with the extreme values of radius of gyration were selected. Analysis of provided MOHCAseq data resulted in identification of contacts between regions nt 9–10 : 26–29 : 49–52 and nt 16–18 : 28–31. In order to force preservation of those contacts in the resultant 3D model with the lowest radius of gyration, the three-way junction 4-UGAC-7; 20-GCGG-23; 55-CA-56 was manually adjusted in PyMol. This 3D motif was used as a user-defined element during 3D model assembly (Antczak et al. 2016). 3D models predicted by RNAComposer are characterized by very good local agreement with the reference structure in regard to the E-loop motif and 10 nt apical loop, and are therefore ranked within the top five 3D models within the MCQ category (Zok et al. 2014) and all INF-based categories (Supplemental Fig. S26).

#### Puzzle 7

The RNA secondary structure was adjusted based on provided experimental data, and base-pairing between regions 27–35 and 90–97 was anticipated from this analysis. Due to the difficulties in prediction of 3D structures preserving mentioned base-pairing interactions and appropriate total energy score, hundreds of various 3D models were generated. The five most diverse 3D models were finally submitted. Since interactions that occur between 27–34 and 90–97 are inter- rather than intramolecular (Suslov et al. 2015), applied experimental data resulted in substantially decreased accuracy.

### Bujnicki group

The Bujnicki group used a hybrid modeling strategy (Piatkowski et al. 2016) based on the approach tested in the previous editions of the RNA-Puzzles experiment (Cruz et al. 2012; Miao et al. 2015). If the target sequence exhibited detectable similarity to an RNA with known experimentally determined structure (as happened in the case of Puzzles 4 and 8), the Bujnicki group generated models of the whole molecule or its parts using a template-based (comparative) modeling method ModeRNA (Rother et al. 2011b) or its server version (Rother et al. 2011a). Remodeling of uncertain regions and modeling of RNA molecules that lacked suitable templates relied mostly on template-free folding using the coarse-grained method SimRNA (Boniecki et al. 2016). In the course of the competition, the Bujnicki group experimented with various versions of the SimRNA force field. For instance, in Problems 12, 13, and 14 they tested different variants of the force field, including ones that could be more easily extrapolated to the standard Turner energy rules (Xia et al. 1998), and introduced further concepts from polymer chemistry (Flory 1969). This is an ongoing project wherein improvements are being continually added. In the course of the experiment the Bujnicki group has also introduced a completely automated version of the program available as the SimRNAweb web server (Magnus et al. 2016). Wherever available, template-free folding was aided by spatial restraints obtained from computational predictions (e.g., information on the secondary structure or orientation of helices in junctions) and from data identified in the literature or made available in the course of the RNA-Puzzles experiment by the Das group. For high-resolution refinement of models, the Bujnicki group used the QRNAS method (J Stasiewicz and JM Bujnicki, unpubl.) that extends the AMBER force field with energy terms explicitly modeling hydrogen bonds, idealizes base pair planarity, and regularizes the backbone conformation.

As in previous modeling exercises, human intervention was relatively extensive. Most of the time was devoted to searching for additional information related to target RNA sequences and discussions within the group. The time used for alignment preparation and for selection of models for submission varied greatly depending on the difficulty of the problem. The time used for template-based modeling was negligible. Time required for modeling with SimRNA ranged from one to a few days per target (depending on the size of the sequence modeled in the template-free mode), and the final refinement was typically run overnight. In addition, the introduction of various restraints often required extensive time developing additional scripts to handle the unique problems inherent in each Puzzle.

The Bujnicki group also explored a completely new way of 3D geometry prediction for RNA molecules. This project was started by M. Magnus during his internship at Stanford with R. Das and was inspired by a method to combine structure prediction runs across diverse homologs, as is carried out in protein structure prediction (Bonneau et al. 2001). Based on the observation that sequences from the same RNA family fold into similar structures, the Bujnicki group explored the possibility that a similar process can be observed in computational modeling and could be used to detect the global helical arrangements for a given target sequence based on the arrangements within a subset of homologs. The proposed method explores the use of multiple sequence alignment information and parallel modeling of RNA homologs to improve 3D structure prediction over modeling of single RNA sequences. To build a structural model of the target sequence, a multistep modeling process is performed. First, for the target sequence, a subset of homologous sequences is selected using the Rfam database. Subsequently, independent folding simulations are carried out for these homologs; in this series of RNA-Puzzles experiments the Bujnicki group used ROSETTA/FARNA (Das and Baker 2007), but in principle any template-free folding method can be used. Structural fragments corresponding to the evolutionarily conserved regions (in particular helices)—determined from the alignment—are extracted from all obtained models and clustered to identify the most common structural arrangement. The Bujnicki group also explored a way to constrain the simulation by keeping the conserved residues identified by the alignment as being in close spatial proximity. The approach was used in modeling of Puzzles 13 and 14. In a blind prediction of Puzzle 13, a model obtained with this methodology was second, and in a Puzzle 14 (bound form), one model was best in terms of the RMSD to the reference structure. This approach is now under further systematic tests in preparation to make it automated and available for the community. The current version of the program, documentation, and input files to solve Puzzles 13 and 14 can be accessed under a github repository at https://github.com/mmagnus/EvoClustRNA.

#### Puzzle 4

For Puzzle 4, the Bujnicki group submitted the structure of the complete RNA–protein complex. The models of both RNA and protein components have been constructed using homology modeling, as suitable templates could be found in both cases. The RNA part was based on a homologous *Thermoanaerobacter tengcongensis* SAM-I riboswitch structure (PDB ID: 3IQP) used as a template. The structure of the YbxF protein was predicted by fold-recognition using the GeneSilico metaserver (Kurowski and Bujnicki 2003), followed by template-based modeling using the L7Ae protein structure (PDB ID: 2FC3) as a template, with MODELLER (Sali and Blundell 1993). RNA–protein docking was performed using a procedure that in the meantime has been automated and now is available via the NPDock web server (Tuszynska et al. 2015). The models turned out to be quite accurate (the best one had 3.99 Å RMSD to the reference structure).

#### Puzzle 7

This RNA structure was predicted in a template-free mode. The Bujnicki group knew from the problem description that the target sequence crystallized as a dimer. Following this clue, the Bujnicki group generated two types of dimers, all based on an initial model of a monomer, generated with restraints on secondary structure. Two types of dimers were proposed: one type obtained by manual docking of two copies of the monomeric structure, and another obtained by domain swapping. The domain-swapping hypothesis was later found to be incorrect. While the experimentally determined structure forms a dimer using intermolecular base pairs between residues 31–33 in one chain and 92–94 (numbering according to the target sequence) in the other chain, the Bujnicki group assumed that these residues form intramolecular pairs within the monomer, leading to a distortion of the global architecture, despite otherwise correct predictions of other structural elements.

#### Puzzle 8

This RNA structure was predicted by template-based modeling, based on the structure of the *S-*adenosylmethionine riboswitch regulatory mRNA element (PDB ID: 2GIS), followed by template-free refinement with restraints on secondary structure, including a pseudoknot. The models turned out to be quite accurate (the best one had 6.71 Å RMSD to the reference structure).

#### Puzzle 12

The structure was modeled in a template-free mode, using restraints on secondary structure derived from a search of Rfam and other sources for homologous sequences with known structures. Simulations were run in two versions: with and without the predicted pseudoknot. The Bujnicki group also introduced some mutate-and-map restraints based upon visual estimation of intensity and a very simple in-house program for reading the mutate-and-map data provided by the Das laboratory. Based on information provided for this problem, the Bujnicki group assumed there should be a binding pocket for cdiAMP and they filtered the models obtained based on the proximity of conserved residues. This turned out to be an error, as the structure turned out to have two binding pockets. Nonetheless, the Bujnicki group was able to obtain models with globally correct architecture, despite local inaccuracies in secondary structure and wrong topology of the chain in one of the regions (between P2 and P3 helices). Other than that, the biggest challenge was to get the coaxial stacking of helices right. In this exercise the Bujnicki group ran out of time and did not subject the models to refinement with QRNAS before the deadline, which led to the high steric clashes in the models.

#### Puzzle 13

The structure was generated using template-free modeling, with restraints on the secondary structure derived from an Rfam alignment (plf family), and the Bujnicki group included the pseudoknot as described by the Breaker group (Kim et al. 2015). They obtained models that had generally correct secondary structure and global architecture, although some models exhibited an incorrect topology (e.g., a triple helix) because they over-interpreted the experimental data, leading to excessive packing. After publishing the results, the Bujnicki group found that SimRNA run without restraints based on the experimental data gave better models (<6 Å RMSD to the reference structure).

#### Puzzle 14

This structure was modeled using a hybrid approach, by combining template-based modeling for individual structural fragments, followed by global folding with restraints. As templates, the Bujnicki group used the E-loop motif from the structure of *H. marismortui* ribosome (PDB ID: 1JJ2) and of the sarcin–ricin loop motif (PDB ID: 1Q9A). Models of these fragments were used as a source of distance restraints for SimRNA, derived using an in-house-developed tool. The Bujnicki group also used restraints on the secondary structure of the whole molecule derived from an Rfam alignment (GlnA family). Further, they used the CARTAJ method (Lamiable et al. 2012) to predict the architecture of the junction and generated restraints to enforce coaxial stacking of helical regions. Further, restraints on individual base pairs were added based on our interpretation of additional information available for the free and bound forms. A separate set of folding simulations was carried out with additional information from the mutate-and-map data and MOHCA data provided by the Das laboratory (to automatically generate the SimRNA restraints using this type of information, the Bujnicki group used our in-house tools developed by W. Dawson). The restraint on the G23–C60 base pair predicted for the bound form turned out to be correct and the Bujnicki group obtained very accurate models. The best prediction of our group was generated using M. Magnus’ “evolution-based modeling” and it was the best model among all groups (in terms of RMSD) (Magnus et al. 2016). However, due to mispredicting a G22–U61 base pair (which is not present in the free form), the Bujnicki group failed to predict the large conformational change between the bound and the free form, resulting in globally incorrect models of the free form.

### Chen group

The Chen group used a hierarchical approach to predict RNA 3D structure from the sequence (Xu et al. 2014). For a given RNA sequence, they first predict the secondary structure from the free energy landscape using the Vfold2D model. Then they predict the all-atom 3D structures using the Vfold3D model, based on the predicted 2D structure. In general, the accuracy of the 3D structure prediction mainly relies on the accurate prediction of the 2D structure, since the Vfold3D model uses the 2D motif to search for the templates to build the all-atom 3D structures. A slight change in 2D structure, such as the closing or opening of a single loop-closing base pair, may lead to a different 3D template for the loop structure in the predicted 3D structures.

In order to further increase the accuracy of RNA 2D structure prediction, they also applied Rfam (Burge et al. 2013) to identify the possible conserved base pairs and used the most conserved base pair information as a constraint to the Vfold2D algorithm to predict 2D structures. They found that, for the cases of which Rfam results are available (Puzzles 8, 12, 13, and 14), the 2D structures could be correctly determined by the hybrid method (combining the sequence analysis and the Vfold2D free energy-based model). For structures containing no cross-linked base pairs, such as Puzzles 8 and 14, the Vfold2D obtained the same/similar predictions as the results from the hybrid method. However, for the cases with cross-linked base pairs, such as Puzzles 12 and 13, the Rfam data can indeed increase the accuracy for the 2D structure prediction. For Puzzle 7, which did not have Rfam data, the Vfold2D gave ∼90% (54/60 bps) prediction of the native base pairs (see also the INF all/wc/nwc result in the result summary tables).

For the 3D template-based structure prediction algorithms, such as Vfold3D, a critical limitation is that not all motifs have proper templates in the template database built from the known PDB database. They used the Rosetta (assembly) package to sample the all-atom motif 3D structures for the motifs without any templates. The top five cluster centers (centroid structures) were selected for further structure assembly in the Vfold3D to build 3D structures of the whole RNA. For example, the all-atom structures of one of the three-way junctions in Puzzle 12 were generated by the Rosetta package. They used the A-form helix to build the all-atom helix structures. The treatment of the A-form helices, which have small differences from the real (slightly distorted) all-atom 3D helix structures, could also result in notable structural differences in the global fold (Xu and Chen 2015).

In summary, the computation involved two steps: the prediction of the 2D structure and the construction of the 3D structure, followed by the AMBER minimization. They manually incorporated the constraints from the Rfam results into the Vfold2D model for 2D structure predictions. In general, the prediction can be completed within 5 h. However, if the Rosetta package is used to generate the centroid motif structures, it may take 2 d to sample 50,000 structures using 8 CPUs (Intel Core i7-2600 CPU at 3.40 GHz). Their predictions did not take into account the effect of the ligand molecules on the structure of RNA.

### Das group

The Das group created 3D models through homology modeling and de novo modeling (fragment assembly of RNA with full-atom refinement) methods in the Rosetta modeling package, as described in Cheng et al. (2015b). By the time of later targets (puzzles 12–14), these runs became largely automated after identifying secondary structure, possible templates, and experimentally guided constraints, as noted below:

#### Puzzle 4

This protein/RNA modeling provided an opportunity to test high-resolution stepwise assembly of the RNA-contacting protein loop and is described in Das (2013). The helix extension P3ext was modeled in an over-compressed conformation due to use of an incorrect force field in Rosetta's rna_helix.py; after the crystal structure was released, this problem was identified and then traced by Molprobity analysis. The repulsion between molecular hydrogens was too weak in Rosetta. The effect was corrected for later targets and in other Rosetta RNA applications (Chou et al. 2016). Independently, protein modeling work in Rosetta discovered a similar issue (Park et al. 2016).

#### Puzzle 7

The RNA was modeled as a monomer except for one run as a dimer that gave the final model, assuming cross-dimer swapping of P1, a possibility noted in Ouellet et al. (2009) that turned out to be the case in the crystal structure. However, problems were apparent during modeling due to mismatches of which regions were modeled as buried compared to regions found to be protected in hydroxyl radical footprinting experiments (Supplemental Fig. S18). Furthermore, an apparent signal for a contact between A652 and A726 in mutate-and-map measurements (Supplemental Fig. S18), which turned out to be correct, could not be reconciled with our models. After release of the crystal structure, these inconsistencies with experiments were traced to using models of the P2–P3–P6 junction enforcing P3–P6 coaxial stacking (Lafontaine et al. 2001), which turned out to be incorrect; modeling with less human bias and more automated use of experimental data might have improved accuracy.

#### Puzzle 8

This RNA was modeled based on previously detected homology to SAM I in the core, secondary structure confirmed by mutate-and-map experiments, and de novo building of peripheral tertiary contacts, essentially as described in Cheng et al. (2015b).

#### Puzzle 12

Although mutate-and-map experiments gave accurate secondary structures, this RNA was modeled with an incorrect extra stem pair P4, based on prior literature modeling, which precluded agreement in the “bubble”region. The Rosetta modeling included modeling of the two cdiAMP ligands, with possible binding partners tested among conserved guanosines in the riboswitch, and ranking based on structural convergence of the RNA. MOHCA-seq, which discovers tertiary proximities, was applied too late in the modeling to influence the structure prediction, but post facto comparisons suggest inclusion of MOHCA-seq would have improved accuracy (Supplemental Fig. S19). See Tian and Das (2016) for further discussion of how more automated use of experiments would likely have improved accuracy.

#### Puzzle 13

The most accurate model came from use of a strong MOHCA-seq constraint connecting A28 to the molecule's core (Supplemental Fig. S20). Rosetta modeling included ZMP ligands with binding partners posited to be conserved uracils in the riboswitch. This target also promoted the development of a *clustix*-Rosetta method to combine structure prediction runs across diverse homologs, as is carried out in protein structure prediction (Bonneau et al. 2001), in a collaborative effort with Magnus and Bujnicki, as described above (Magnus et al. 2016).

#### Puzzle 14

This modeling benefited from recognition of the loop E module, but otherwise involved standard FARFAR or stepwise modeling in Rosetta. Experimental data for the bound state, which turned out to be rather extended, did not reveal any proximity to aid modeling.

### Ding group

RNA structure predictions by the Ding group try to incorporate various structural data (both secondary and tertiary structures) available in the literature (e.g., bioinformatics, hydroxyl radical foot printing, and in-line probing) or provided by the organizer (e.g., SHAPE and proximity-mapping from the Das group). Briefly, for each given sequence, the database search with Rfam (Burge et al. 2013; Daub et al. 2015) is first performed to determine the RNA molecule's identity, function, homology, and multiple sequence alignment (MSA). In the case of Puzzle 4, where structures of homologous RNA sequences are experimentally known (e.g., PDB ID: 2GIS), a homology modeling approach with multiscale discrete molecular dynamics (DMD) modeling (Ding et al. 2008a) is adopted. With the coarse-grained RNA model, sequence variations such as mutations, insertions, and deletions are straightforwardly carried out. The evolutionarily conserved structural elements are constrained with respect to the corresponding experimental structures in the coarse-grained DMD simulations. The coarse-grained structural models with low free energies during the course of DMD simulations are collected and subjected to clustering analysis to select representative model structures (i.e., 10 models). For each coarse-grained structural model, all-atom reconstruction using the Medusa force field (Ding et al. 2008b) is performed as described in iFoldRNA (Sharma et al. 2008).

In other cases without known homology structures, a hierarchical modeling approach is applied. At the coarse-grained modeling stage, non-pseudoknot secondary structures and then the pseudoknots are imposed stepwise using base pair constraints (Gherghe et al. 2009). The base pairs obtained from MSA are used if the RNA sequence is annotated in the Rfam database. Otherwise, base pairs derived from secondary structure predictions (with SHAPE if available) are used. Usually, 10 independent simulations are performed with different initial conditions. Next, the tertiary structural information including chemical probing by hydroxyl radical footprinting and in-line probing and proximity mapping are incorporated. The solvent accessibility information can be obtained from the chemical probing experiments (Ding et al. 2012). Since the chemical probing data from the literature are often nonquantitative, the nucleotides are simply categorized as exposed, buried, and intermediate based on visual inspection of published plots. For the proximity-mapping data (e.g., the MOHCA data), the Ding group develops a weak bias potential model to drive DMD simulations toward the experimentally consistent state. Attractive potentials are assigned between nucleotide pairs with the MOHCA intensity, *I*, larger than the average value, <*I*>; and the corresponding attraction strength, ɛ = −*K*_*B*_*T*ln(*I*/〈*I*〉)/2, where *K_B_T* is assumed 0.6 kcal/mol for the temperature *T* = 300 K, and *K*_*B*_ denotes the Boltzmann constant. A stepwise potential function as used previously (Gherghe et al. 2009) with three attractive steps between 30 and 50 Å is used. In the case of Puzzle 8, proximity constraints can be inferred from nucleotides known to bind SAM. The Ding group did not explicitly model the ligand, but assigned pairwise proximity constraints between 3–26, 8–25, and 48–69 for the SAM-I/IV riboswitch. All available tertiary structure information is included in the coarse-grained DMD simulations. For each of the ten independent simulations, the lowest free energy structure is obtained and the corresponding all-atom representation is reconstructed accordingly. For the above RNA structure prediction procedure, the manual steps include database and literature searches, the preparation of base pair inputs, and chemical probe data. The rest of the procedures are automated.

### Dokholyan and Weeks groups

The Dokholyan group participated in Puzzles 4, 7, 8, 12, and 13; Puzzle 12 was undertaken in collaboration with the Weeks group. Structure modeling was performed using discrete molecular dynamics (DMD) simulations (Dokholyan et al. 1998; Proctor et al. 2011). The Dokholyan group's structure prediction pipeline comprises sequential steps. First, simulations are performed using coarse-grained RNA geometry and a simplified force field (Ding et al. 2008a). Each nucleotide is represented by three pseudoatoms corresponding to the phosphate group, sugar, and nucleobase. The connectivity and the local geometry of the RNA chain is supported by restraints on bond lengths, bond angles, and dihedral angles optimized using a set of high-resolution RNA structures. The model includes potentials for base-pairing (A–U, G–C, and U–G), base-stacking, short-range phosphate–phosphate repulsion, and hydrophobic interactions (Ding et al. 2008a). Additional constraints can account for putative secondary structure, tertiary contacts, or ligand binding pockets. Replica exchange simulations are used to enhance sampling of the RNA conformational space. Next, the lowest (10% of) energy structures are selected from the replica exchange simulations and clustered hierarchically to identify the dominant state among the lowest energy ensemble. Finally, the centroids of the most populated clusters are selected for all-atom reconstruction. All-atom reconstruction is adapted from protein modeling (Shirvanyants et al. 2012). The DMD-direct modeling process is fully automated (Krokhotin et al. 2015). For Puzzle 4, the Dokholyan group built the initial tertiary structure of the target RNA based on the structure of a homologous sequence (PDB ID: 3IQP). This structure was converted to a coarse-grained model, run through DMD simulations, and converted back to an all-atom model for refinement.

In all other puzzles, the Dokholyan group performed simulations starting from extended RNA molecules providing a secondary structure as restraints. In Puzzles 7, 8, and 13, the secondary structure was predicted using Mfold (Zuker 2003). For Puzzle 12, the Dokholyan group first searched for the sequence and obtained a single hit from *T. tengcongensis*, 5′ of the OppA9 gene, annotated as an ABC-type protein with nickel transporting ability. SHAPE-MaP data were then obtained with and without the (incorrectly) presumed ligand Ni^2+^. Despite using the incorrect ligand, significant changes in SHAPE reactivity were observed as a function of Ni^2+^. SHAPE-MaP-directed modeling (Siegfried et al. 2014) using ShapeKnots (Hajdin et al. 2013) resulted in a structure with a correctly predicted pseudoknot and with overall sensitivity and positive prediction values of 82.3% and 69%, respectively. This minimum free energy secondary structure was used as the basis for submissions 1 and 2. After the Das group released their MOHCA-seq (Cheng et al. 2015a) proximity data, the Dokholyan group selected an alternative secondary structure (with improved 94.3% sensitivity and 80.5% positive predictive value). Despite the high accuracy of the secondary structure model, the accuracy of the predicted tertiary structure was only moderate (submission 3, RMSD ∼19.8Å). Ultimately, they were challenged by their inability to correctly predict the ligand and by the short turnaround time for the puzzle.

### Xiao group

In this round of RNA-Puzzles, the Xiao group participated in Puzzles 12 and 13. In each case, Mfold (Zuker et al. 1999) was used to predict 2D structure and 3dRNA (Zhao et al. 2012) to predict 3D structures of the given sequence, without using any experimental data. The 2D structures used were “…..((((..(((….)))(((((((.((((….)))).(((..(((((((….))))))).((((….))))))).)))….))))))))…(((((… …..)).)))……” in Puzzle 12 and “…((((…..))))..[[[[[[[……((((((…..))))))……..(((.)))]]]].]]]” in Puzzle 13. It is noted that the 2D structures used by them are different from the native ones and so the predicted WC pairs are low in accuracy (group Xiao in Supplemental Table S3). The Xiao group assembled a preliminary structure based on the 2D structure using our updated template library of secondary structure elements (SSE), and then repeatedly replaced the template of a randomly selected SSE to produce an ensemble of 1000 candidates. These candidates were clustered into 10 classes and scored by 3dRNAscore (Wang et al. 2015) to pick out the best scoring structure from each class. All the steps are fully automated and done quickly. It is noted that one of the 10 structures submitted for Puzzle 13 (number 1 of group Xiao in Supplemental Table S4) was optimized using molecular dynamics for 80 ns. 3dRNA could not yet consider the effect of ligands.

### Discussion and conclusions

With this round of RNA-Puzzles, we started to test and evaluate several aspects of RNA 3D structure prediction aside from structural similarity compared to native crystal structures. We assessed the predictions in template-based structures, homology-derived structures, and fully de novo structures. The targets were quite diverse and included single-ligand binding and double-ligand binding riboswitches, a structure that showed notable ligand-induced conformational changes, and a large ribozyme. We also began to assess more carefully the influence of fast-track chemical mapping data.

For template-based predictions, current modeling methods have achieved a consistently high level of accuracy: It is possible to model nearly all the structural details when a clear homolog can be identified. For the SAM riboswitch aptamer bound to YbxF protein and the SAM I/IV riboswitch aptamer, it was possible to predict a structure with known close or rather distant relatives to explore functional characteristics in structure, including protein–RNA binding interfaces and previously unseen peripheral RNA tertiary domains. Particularly for the SAM I/IV RNA, structural clues were effectively derived for modeling despite quite distant homology, as illustrated by the accuracy of the final structures. In addition, the ligand binding sites were readily inferred via homology.

For targets without homology to previously solved structures (riboswitch aptamers for ZMP, for two c-di-AMP, for L-glutamine, and for the Varkud satellite ribozyme), the modeling quality depended on the size of the molecule. For the smaller two of these RNAs—the ZMP and L-glutamine riboswitch aptamers—blind models with ∼6 Å accuracy achieved striking recovery of the global folds of these targets and, encouragingly, were submitted by several independent groups. In the case of the L-glutamine riboswitch, ligand binding induced a major conformational change, which nevertheless was captured among models in the submissions. While promising, this result comes with a caveat—no group was able to rank these accurate models as their top-choice predictions. Worse predictions (10 Å and 20 Å best-case RMSDs) were made for the two larger de novo modeling targets, the *ydaO* c-di-AMP riboswitch and the VS ribozyme. Nevertheless, for the *ydaO* case, the overall global fold was correct in models from several groups. The size, dimerization, and a “rule-violating” junction of the VS ribozyme appear to explain the difficulty of modeling the structure of that RNA.

In all cases, modelers reported that fast-track experiments helped in defining the secondary structures and, in some cases, topological orientations of helices, similar to our prior report (Miao et al. 2015). We also began trying to assess more rigorously the help of these experimental data. We evaluated predictions made before and after release of experimental data for one target herein, the glutamine riboswitch. While systematic improvement from experimental data appeared to occur for the ligand-free state, we did not detect similar improvement for the more extended ligand-bound state; further pre- and post-experiment comparisons will be important in future rounds of RNA-Puzzles. It may be possible to also test whether other sorts of experimental data might guide blind modeling; the CASP protein structure prediction trials now include some targets for which small angle X-ray scattering, crosslinking, or NMR chemical shift data are provided.

The large number of riboswitch aptamer structures allowed a detailed assessment of prediction of ligand binding sites and fine orientation. While ligand modeling appeared quite accurate for the SAM riboswitch, whose structure turned out to be clearly similar to previously solved structures, modeling of ligand binding sites achieved no better than nucleotide resolution for the ZMP and *ydaO* c-di-AMP cases. No ligand binding predictions were submitted for glutamine. In each of these cases, evolutionary conservation, but not the available chemical mapping data, gave weak clues as to ligand binding sites. Modeling these functionally important interactions may require special knowledge of the ligand binding and nontrivial changes to the RNA structure. Further biological insights or advances in high-resolution structure prediction will be needed to improve predictive understanding of ligand binding from nanometer-level resolution to the Angstrom-level resolution attained by crystallography.

As described in the previous round RNA-Puzzles II, prediction of non-Watson–Crick interactions and achievement of acceptable clash scores remain critical areas for improvement. Watson–Crick interactions in some structures have already been perfectly predicted, but non-Watson–Crick interactions are difficult and it is known that they contribute crucially to the structures and interactions. Non-Watson–Crick base pairs are more variable and have different covariation rules than Watson–Crick interactions, and this may be difficult to capture both in experiments and prediction. Moreover, we notice some top ranking models still include a large number of atomic clashes. To optimize the models to a more harmonious state could still be improved in future work. Another point made in round II was the need for assessing automated servers; such testing is occurring with recent puzzles, but crystal structures have not been released for those targets. Server testing will be evaluated in the next round.

It could still be good to develop new assessment criteria to balance the global and local similarities. RMSD may overemphasize the assessment of global similarity of all the atoms. MCQ score provides an alternative to assess the structure in torsion angle space to alleviate the effect of local differences that leads to topological deviation. However, MCQ score is not very discriminative in assessing structure topology. Bond length and bond angles that hardly change may also affect the structure aside from the torsion angle.

For several of the points above—the need for more precise ligand binding positions, for better clash scores, for the identification of non-Watson–Crick interactions, and for more accurate prospective ranking of good solutions—progress may require a distinct category of RNA-Puzzles. Methods for refining ligand binding sites or noncanonical motifs or for evaluating free energy of models based on, e.g., extensive molecular dynamics sampling, cannot currently be tested due to their computational expense. As in CASP, it may be possible to drive progress in this high-resolution refinement problem through a second modeling period for selected RNA-Puzzle targets whose structures have not been publicly released. During this period, the assessors would release the best model submitted in the first modeling period and give suggestions for where and what to refine, including possible ligand binding sites. These RNA-Puzzle-Refinement models would then be separately assessed with higher resolution criteria.

Here, the Puzzles were organized according to their biological functions (riboswitch or ribozyme) as done previously. The set of metrics developed distinguish the different structural features that are correctly or wrongly predicted in each case. However, despite the quality of some models, we are very far from being able to distinguish between a riboswitch or a ribozyme function on the sole basis of the folded structure. Here, we introduced the prediction of ligand binding to a given riboswitch, but the nature of the ligand was revealed. Future challenges should address the predictions of the three-dimensional fold of a riboswitch as well as the nature and binding mode of the target ligand.

## SUPPLEMENTAL MATERIAL

Supplemental material is available for this article.

## Supplementary Material

Supplemental Material
