# Supplementary material for: RNA-Puzzles Round III: 3D RNA structure prediction of five riboswitches and one ribozyme
Source: RNA. 2017 May;23(5):655–72. doi: 10.1261/rna.060368.116 (PMC5393176; doi:10.1261/rna.060368.116)
Supplement: Supplemental Material [file supp_23_5_655__index.html]

RNA-Puzzles Round III: 3D RNA structure prediction of five riboswitches and one ribozyme — Supplemental Material 

# RNA-Puzzles Round III: 3D RNA structure prediction of five riboswitches and one ribozyme

## Supplemental Material

Supplemental Material

- Supp\_Fig\_Legends\_S1-S20.docx
- Supp\_Fig\_S1.jpg
- Supp\_Fig\_S2\_Puzzle4-2.jpg
- Supp\_Fig\_S3\_puzzle4.jpg
- Supp\_Fig\_S4.jpg
- Supp\_Fig\_S5\_Puzzle8-2.jpg
- Supp\_Fig\_S6.jpg
- Supp\_Fig\_S7\_Puzzle12-2.jpg
- Supp\_Fig\_S8.jpg
- Supp\_Fig\_S9\_Puzzle13-2.jpg
- Supp\_Fig\_S10.jpg
- Supp\_Fig\_S11\_Puzzle14-Das2.jpg
- Supp\_Fig\_S12\_Puzzle14-Bound\_Buj2.jpg
- Supp\_Fig\_S13\_Puzzle14-Bound\_chen5.jpg
- Supp\_Fig\_S14.jpg
- Supp\_Fig\_S15\_Puzzle7-2.jpg
- Supp\_Fig\_S16.jpg
- Supp\_Fig\_S17.png
- Supp\_Fig\_S18.png
- Supp\_Fig\_S19.png
- Supp\_Fig\_S20.png
- Supp\_Figs\_S21-S27.docx
- Supp\_Tables\_S1-S7\_RNA\_Puzzles.xlsx
- Supp\_Tables\_S8-S9.docx
