# Supplementary material for: RNA-Puzzles Round III: 3D RNA structure prediction of five riboswitches and one ribozyme
Source: RNA. 2017 May;23(5):655–72. doi: 10.1261/rna.060368.116 (PMC5393176; doi:10.1261/rna.060368.116)
Supplement: Supplemental Material [file supp_060368.116_Supp_Fig_Legends_S1-S20.docx]

***RNA*-Puzzles Round III: 3D RNA structure prediction of five riboswitches and one ribozyme**

Zhichao Miao^1*^, Ryszard W. Adamiak^2^, Maciej Antczak^3^, Robert T. Batey^4^, Alex Becka^5^, Marcin Biesiada^2^, Michał J. Boniecki^6^, Janusz M. Bujnicki^6,7^, Shi-Jie Chen^8^, Clarence Yu Cheng^5^, Fang-Chieh Chou^5^, Adrian R. Ferré-D'Amaré^9^, Rhiju Das^5^, Wayne K. Dawson^6^, Feng Ding^10^, Nikolay V. Dokholyan^11^, Stanisław Dunin-Horkawicz^6^, Caleb Geniesse^5^, Kalli Kappel^5^, Wipapat Kladwang^5^, Andrey Krokhotin^11^, Grzegorz E. Łach^6^, François Major^12^, Thomas H. Mann^5^, Marcin Magnus^5,6^, Katarzyna Pachulska-Wieczorek^2^, Dinshaw J. Patel^13^, [Joseph A. Piccirilli](http://www.sciencedirect.com/science/article/pii/S0022283616303497)^14,15^, Mariusz Popenda^2^, Katarzyna J. Purzycka^2^ , Aiming Ren^13,16^, Greggory M. Rice^17^, Joanna Sarzynska^2^, Marta Szachniuk^3^, Arpit Tandon^11^, Jeremiah J. Trausch^4^, Siqi Tian^5^, Jian Wang^18^, Kevin M. Weeks^17^, Benfeard Williams II^11^,Yi Xiao^18^, Xiaojun Xu^8^, Dong Zhang^8^, Tomasz Zok^3^, Eric Westhof^1*^

**Supplementary figures and tables**

**Fig S1**. Superimposition between 3V7E in green and 3IQR in blue.

**Fig S2**. 2D Heat map of deformation profile of best predicted *Puzzle* 4 model mapped to B factor distribution as histogram on the left.

**Fig S3**. The prediction of SAM binding in *Puzzle* 4. (A) Structure prediction of the SAM binding region. Green is the native structure and blue is model 1 from Das group. (B) The relative orientations of the native and predicted SAM molecules.

**Fig S4**. The prediction of SAM contacts in *Puzzle* 4. (A) Contacts between SAM (magenta) and the riboswitch (green) in crystal structure. (B) Contacts between SAM (blue) and the riboswitch (cyan) in Das model 1.

**Fig S5**. 2D Heat map of deformation profile of best predicted *Puzzle* 8 model mapped to B factor distribution as histogram on the left.

**Fig S6**. SAM binding in SAM-I and SAM-I/IV riboswitches. (A) Superimposition of the SAM-I (blue) and SAM-I/IV (green) riboswitches. (B) Contacts between SAM (deep green) and the SAM-I/IV riboswitch (green). (C) Contacts between SAM (light blue) and SAM-I riboswitch (blue).

**Fig S7**. 2D Heat map of deformation profile of best predicted *Puzzle* 12 model mapped to B factor distribution as histogram on the left.

**Fig S8**. Structure superimposition of the pseudoknot and bubble in *Puzzle* 12. (A) Structure superimposition of pseudoknot between native structure (green) and Ding group model 12 (blue). (B) Structure superimposition of bubble between native structure (green) and Ding group model 12 (blue).

**Fig S9**. 2D Heat map of deformation profile of best predicted *Puzzle* 13 model mapped to B factor distribution as histogram on the left.

**Fig S10**. The prediction of ZMP binding in *Puzzle* 13. (A) Structure prediction of the ZMP binding region. Green is the native structure and magenta is model 1 from Das group. (B) The relative orientation of the ZMP molecules (native as green vs. model 1 from Das group as magenta). (C) Green is the native structure and cyan is model 7 from Das group. (D) The relative orientation of the ZMP molecules (native as green vs. model 7 from Das group as cyan).

**Fig S11**. *Puzzle* 14: 2D Heat map of deformation profile of Das post-experiment model 2.

**Fig S12**. *Puzzle* 14: 2D Heat map of deformation profile of Bujnicki pre-experiment model 2.

**Fig S13**. *Puzzle* 14: 2D Heat map of deformation profile of Chen post-experiment model 2.

**Fig S14**. The prediction of Loop E module in *Puzzle* 14. (A-C) are in Free State while (D-F) are in Bound state. (A) Structure superimposition between native structure (green) and predicted model of Das group post-experiment model 2 (blue). (B) Prediction of Ding group post-experiment model 8. (C) Prediction of Chen group post-experiment model 2. (D) Prediction of Bujnicki group pre-experiment model 2. (E) Prediction of Das group pre-experiment model 6. (F) Prediction of Chen group post-experiment model 6. (a-f) are demonstrations of non-canonical interactions with Leontis-Westhof nomenclature, where circles are interactions with Watson-crick edges, squares are Hoogsteen edge interactions and triangles mean Sugar edge interactions. Grey links show the native interactions should be included in the Loop E module, while black lines show the interactions presented in the predictions.

**Fig S15**. 2D Heat map of deformation profile of best predicted *Puzzle* 7 model mapped to B factor distribution as histogram on the left.

**Fig S16**. The prediction of helices P1-P7 in *Puzzle* 7. (A) Structure superimposition of P1 between native structure (green) and Das group model 1 (blue). (B-G) are the predictions of P2-P7 respectively.

**Fig S17**. Electron density map of nucleotides U67, C68 and G69.

**Fig S18.** Experimental data for *Puzzle* 7. (A) Das group model 1 (left) and 4R4V crystal structure (right) colored by hydroxyl radical footprinting reactivity in 20 mM Mg^2+^. (B) Mutate-and-map data for *Puzzle* 7 with 1M7 modification (RMDB ID: RNAPZ7_1M7_0001). Data for mutant A648U was poor quality and is shown as data for WT RNA. Locations of individual helices are indicated by colored rectangles.

**Fig S19.** Experimental data and predictions for *Puzzle* 12, adapted from Fig 3c and Fig 5e from Tian and Das, 2016. (A) Mutate-and-map data for *Puzzle* 12 with 1M7 modification, probed in the presence of 10 µM c-di-AMP (RMDB ID: RNAPZ12_1M7_0003). Locations of individual helices are indicated by colored rectangles, and yellow arrows mark mutation-induced perturbations that involve disruption of an entire helix rather than release of a single base-pair. (B) Secondary structure models of *Puzzle* 12. The secondary structure assumed by all *RNA-Puzzle* modelers (left) was derived from expert sequence analysis and included an incorrect P4 helix (dark red), while the M^2^-predicted secondary structure (right) correctly rearranged this region. (C) MOHCA-seq proximity map for *Puzzle* 12 (RMDB ID: RNAPZ12_MCA_0000). Cyan contours indicate MOHCA-seq hits corresponding to each secondary structure element. Other contours indicate hits inferred through visual inspection of the proximity map. For ease of visualization, only contours including at least one residue pair with phosphorus-phosphorus (P-P) distance of <45Å in the crystal structure (4QK8) are shown. Coloring of these contours reflect P-P distances of closest approach for residue pairs in the MCM-predicted models (green, <30Å; yellow, 30-45Å; red, >45Å). The sequence positions of secondary structure elements are indicated by the colored axes. (D) MCM predicted model (left) and 4QK8 crystal structure (right), colored according to secondary structure (top) or with cylinders colored as in (C) (bottom). Thick and thin cylinders correspond to strong and weak hits in Cheng et al, 2015, respectively. Gray spheres represent the two c-di-AMP ligands in both the model and crystal structure.

**Fig S20.** Experimental data and predictions for *Puzzle* 13. (A) MOHCA-seq proximity map for *Puzzle* 13 (RMDB ID: RNAPZ13_MCA_0000). Secondary structure elements and other MOHCA-seq hits are annotated by contours as in Fig S19C, and excluding hits between pairs for which at least one residue was in the flexible linker (J-P1/P3). (B) MCM predicted model (Das group model 7, left) and crystal structure (4XWF) (right), colored according to secondary structure (top) or with cylinders colored as in (A) (bottom). Thick and thin cylinders correspond to strong and weak hits used for Rosetta modeling, respectively, excluding hits between pairs for which at least one residue was in J-P1/P3. Gray spheres represent the ZMP ligand in both the model and crystal structure.
