# Supplementary figures and images for: RNA-Puzzles Round III: 3D RNA structure prediction of five riboswitches and one ribozyme
Source: RNA. 2017 May;23(5):655–72. doi: 10.1261/rna.060368.116 (PMC5393176; doi:10.1261/rna.060368.116)

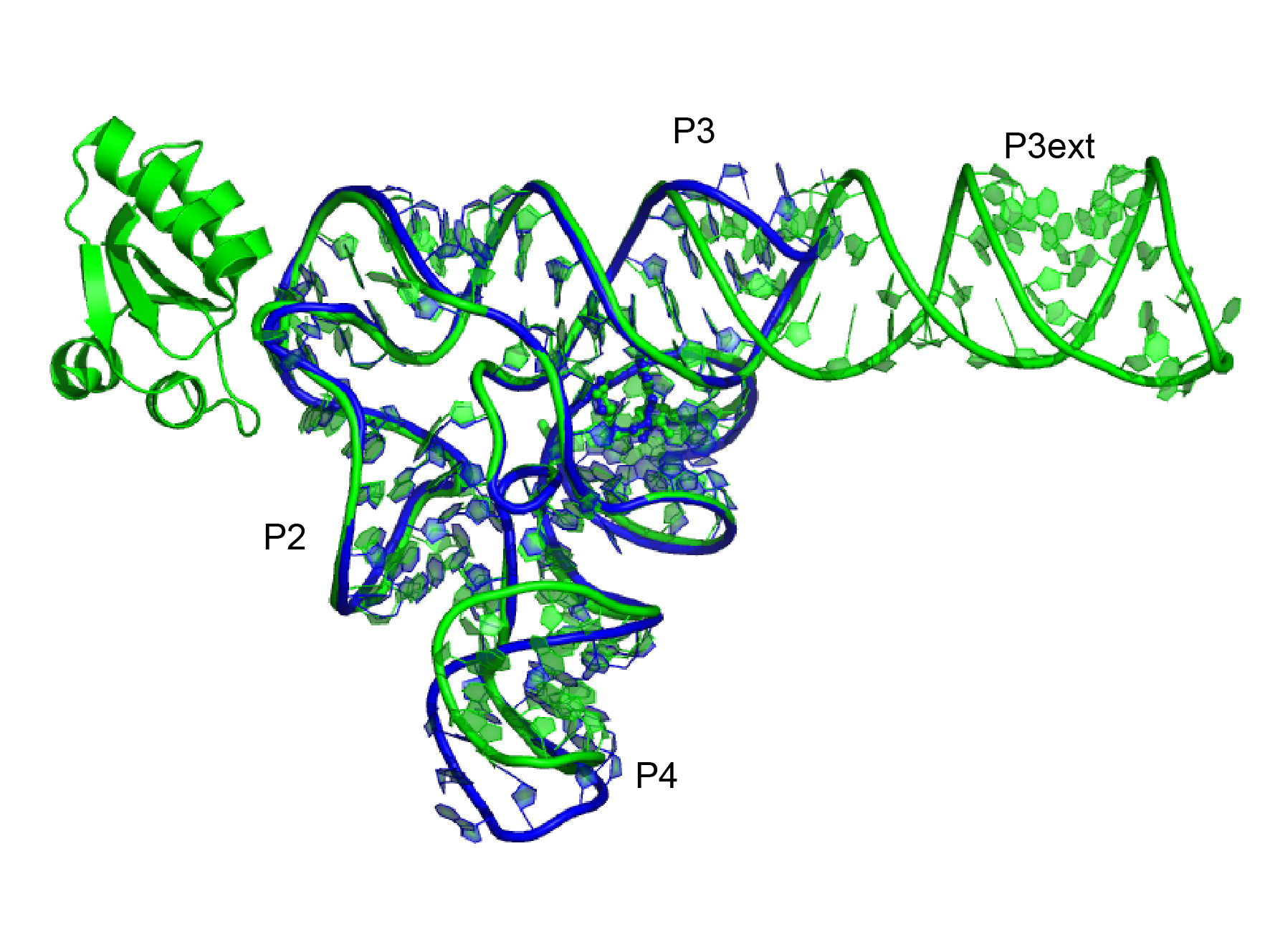

Supplement: Supplemental Material [file supp_060368.116_Supp_Fig_S1.jpg]

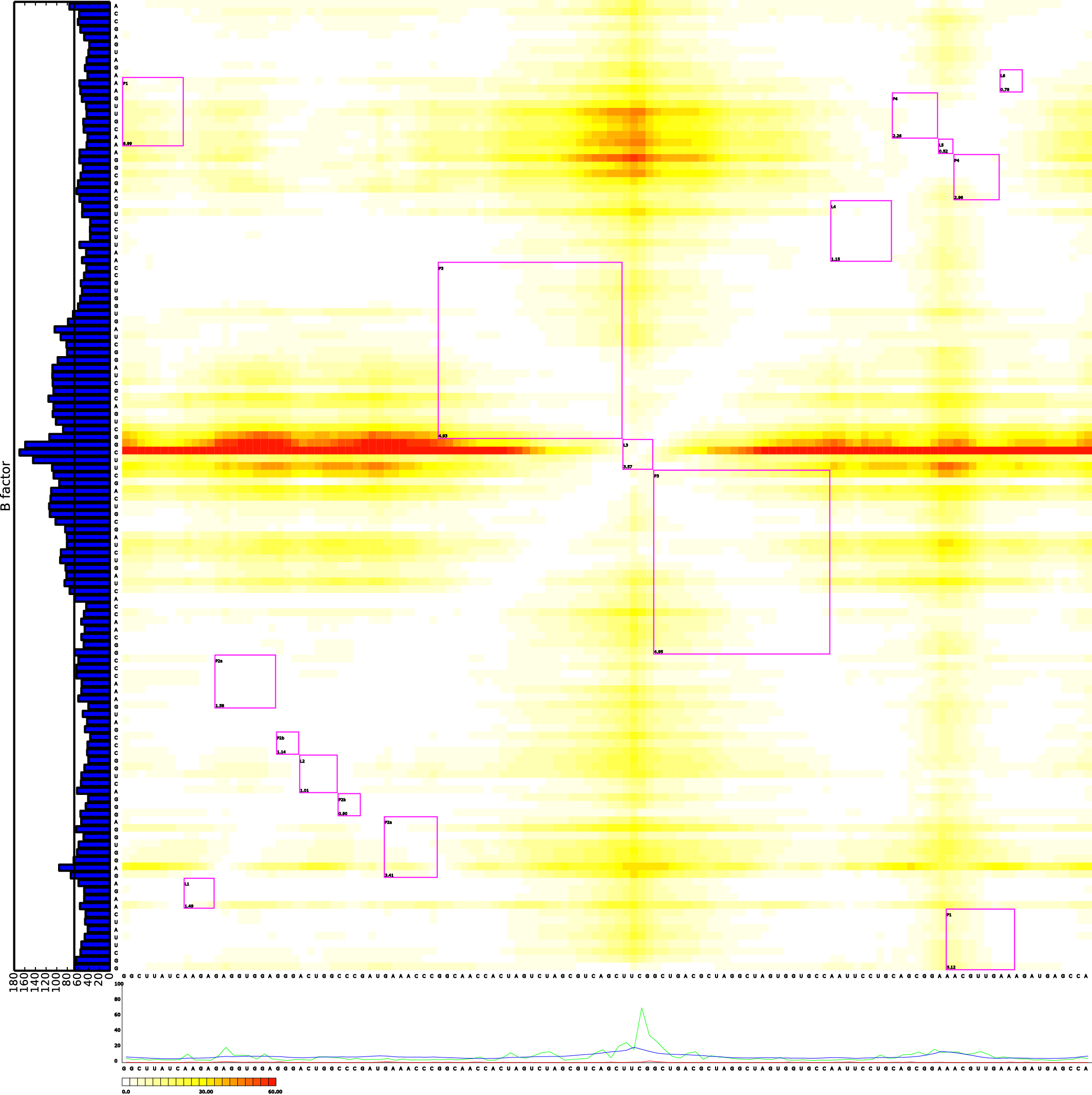

Supplement: Supplemental Material [file supp_060368.116_Supp_Fig_S2_Puzzle4-2.jpg]

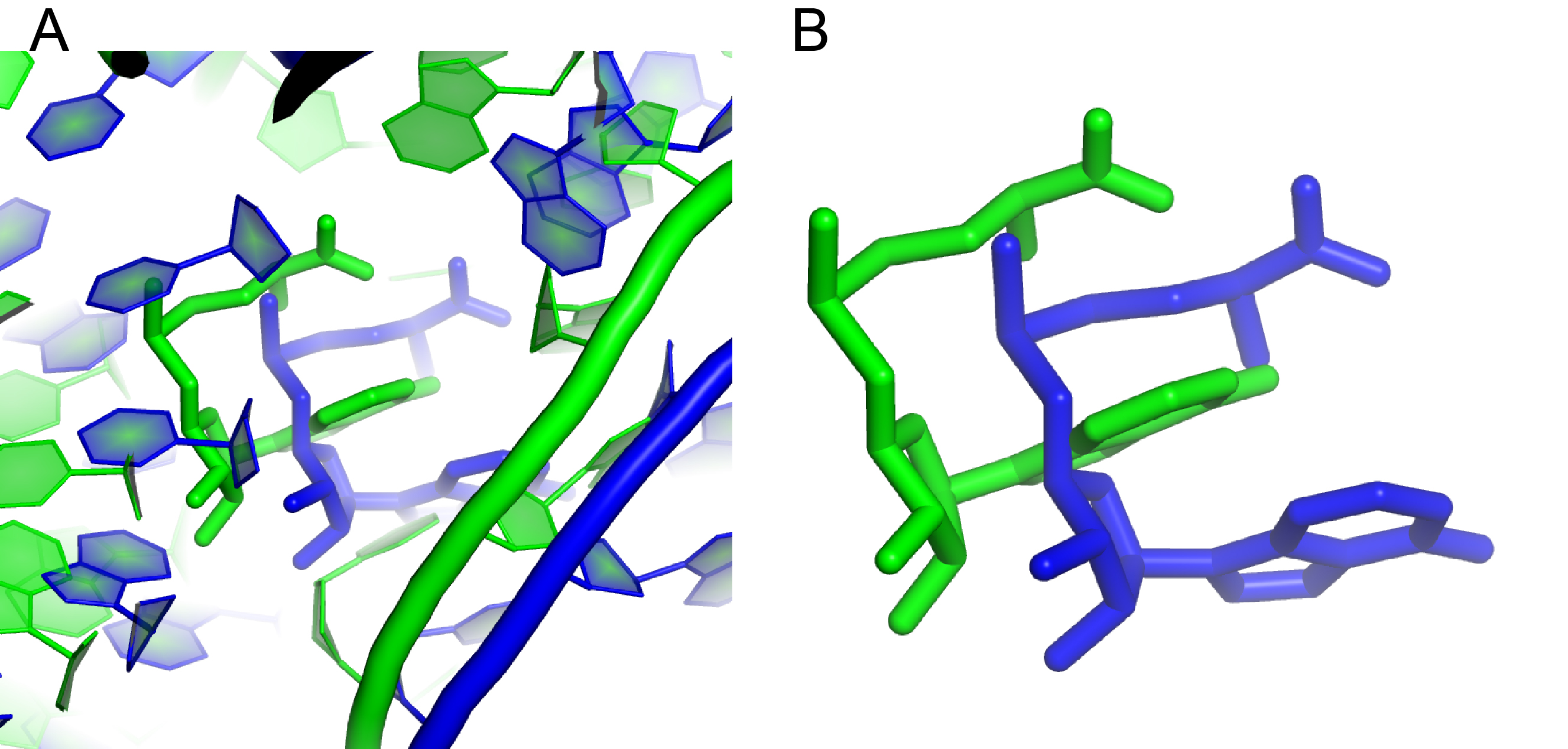

Supplement: Supplemental Material [file supp_060368.116_Supp_Fig_S3_puzzle4.jpg]

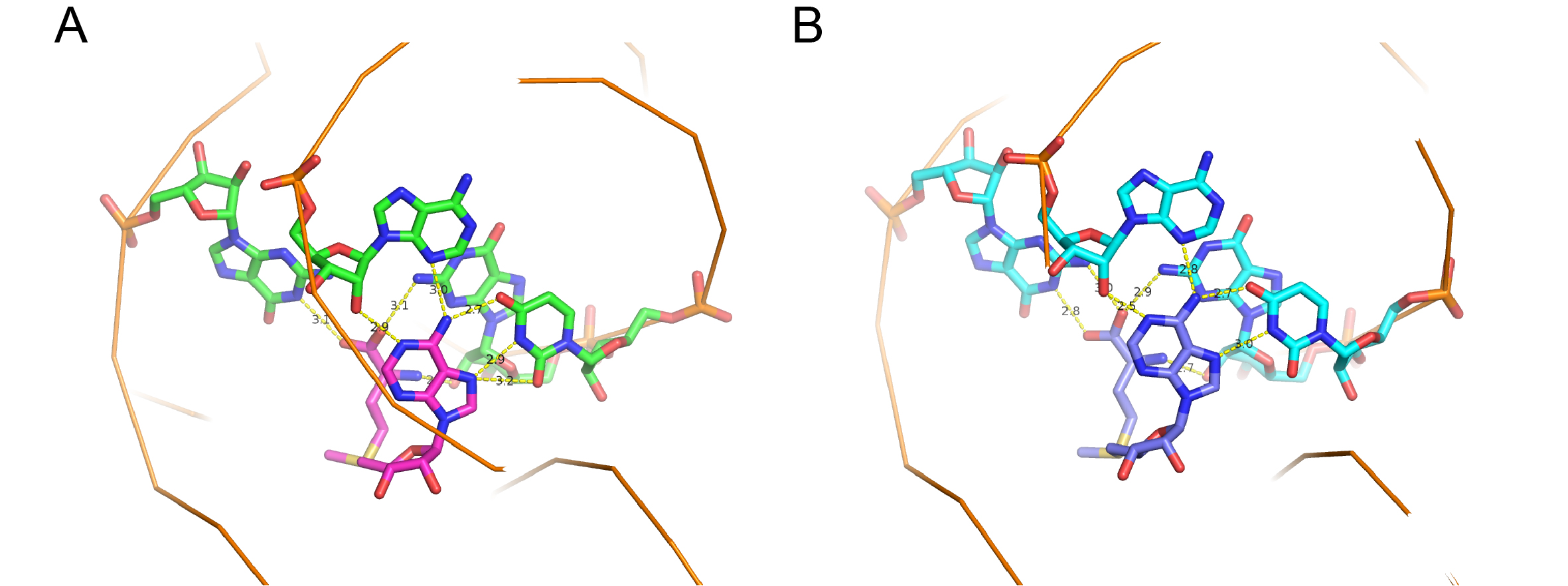

Supplement: Supplemental Material [file supp_060368.116_Supp_Fig_S4.jpg]

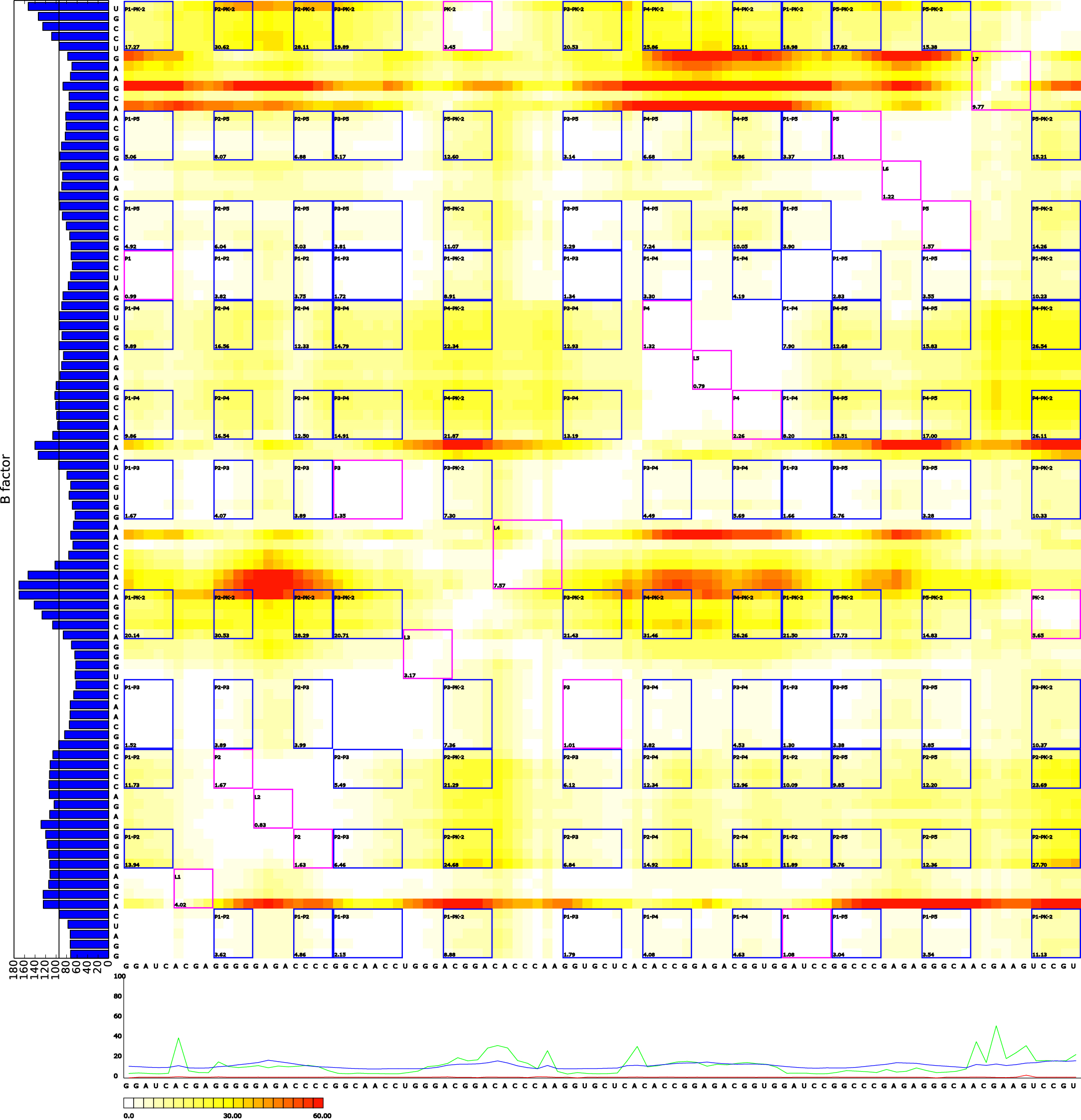

Supplement: Supplemental Material [file supp_060368.116_Supp_Fig_S5_Puzzle8-2.jpg]

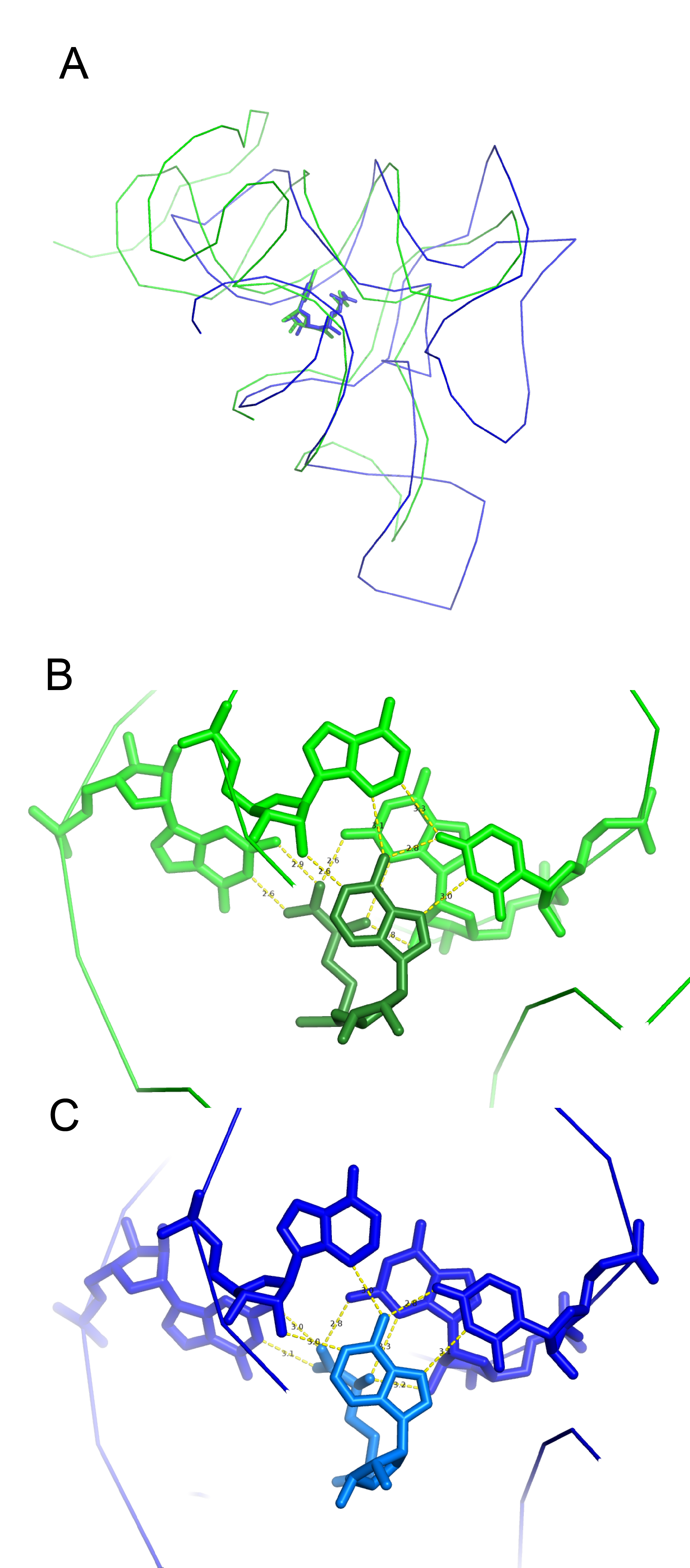

Supplement: Supplemental Material [file supp_060368.116_Supp_Fig_S6.jpg]

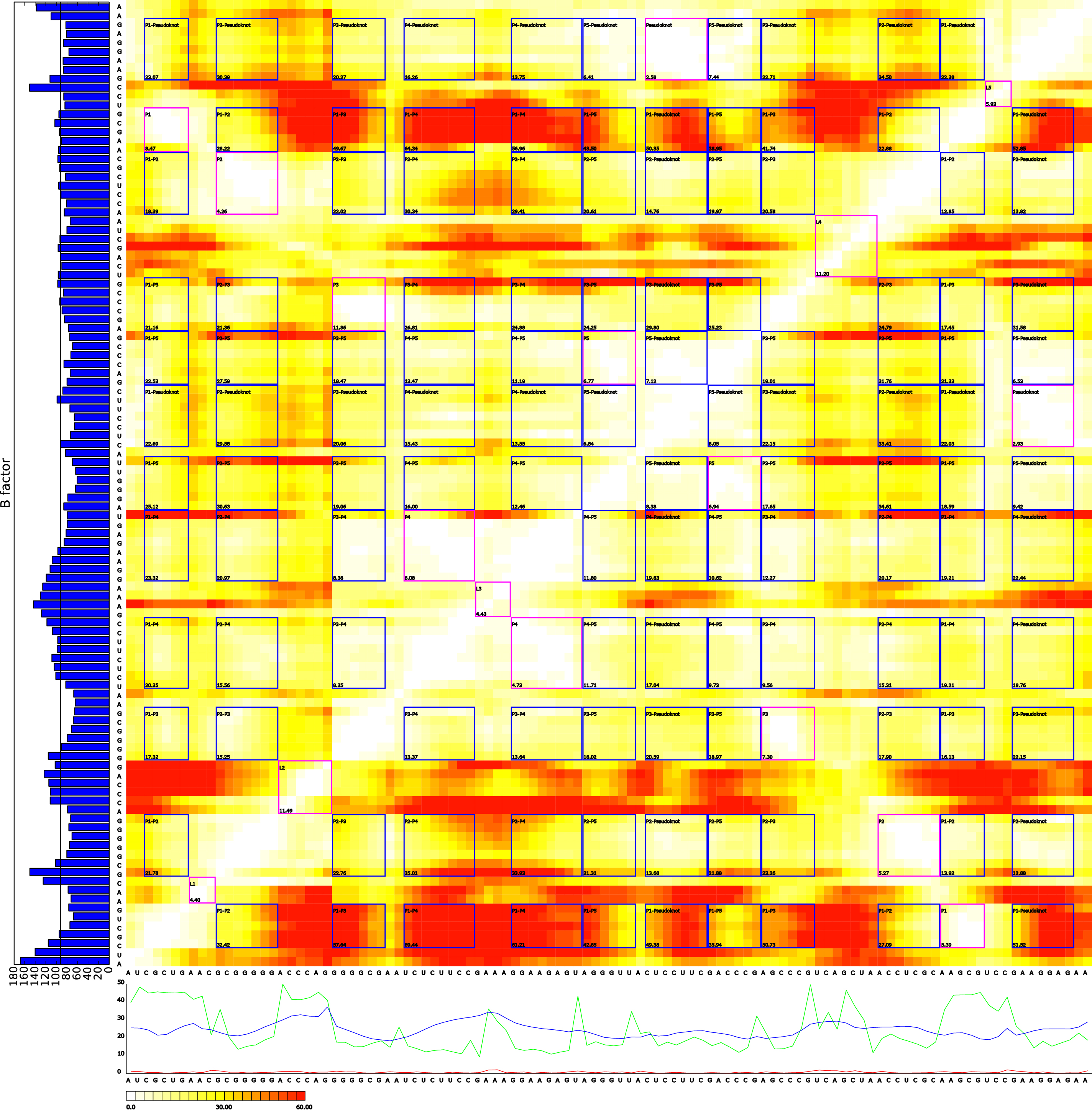

Supplement: Supplemental Material [file supp_060368.116_Supp_Fig_S7_Puzzle12-2.jpg]

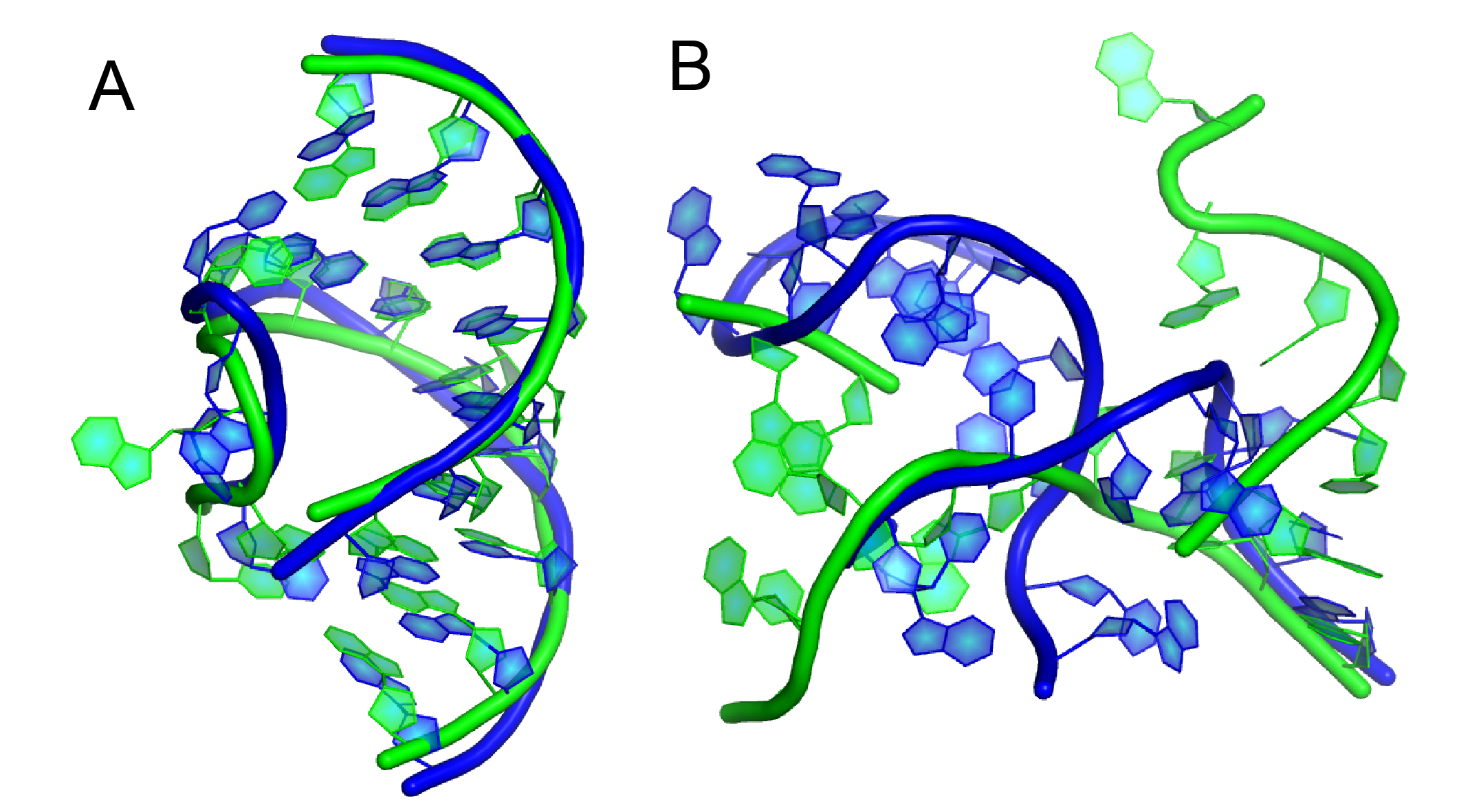

Supplement: Supplemental Material [file supp_060368.116_Supp_Fig_S8.jpg]

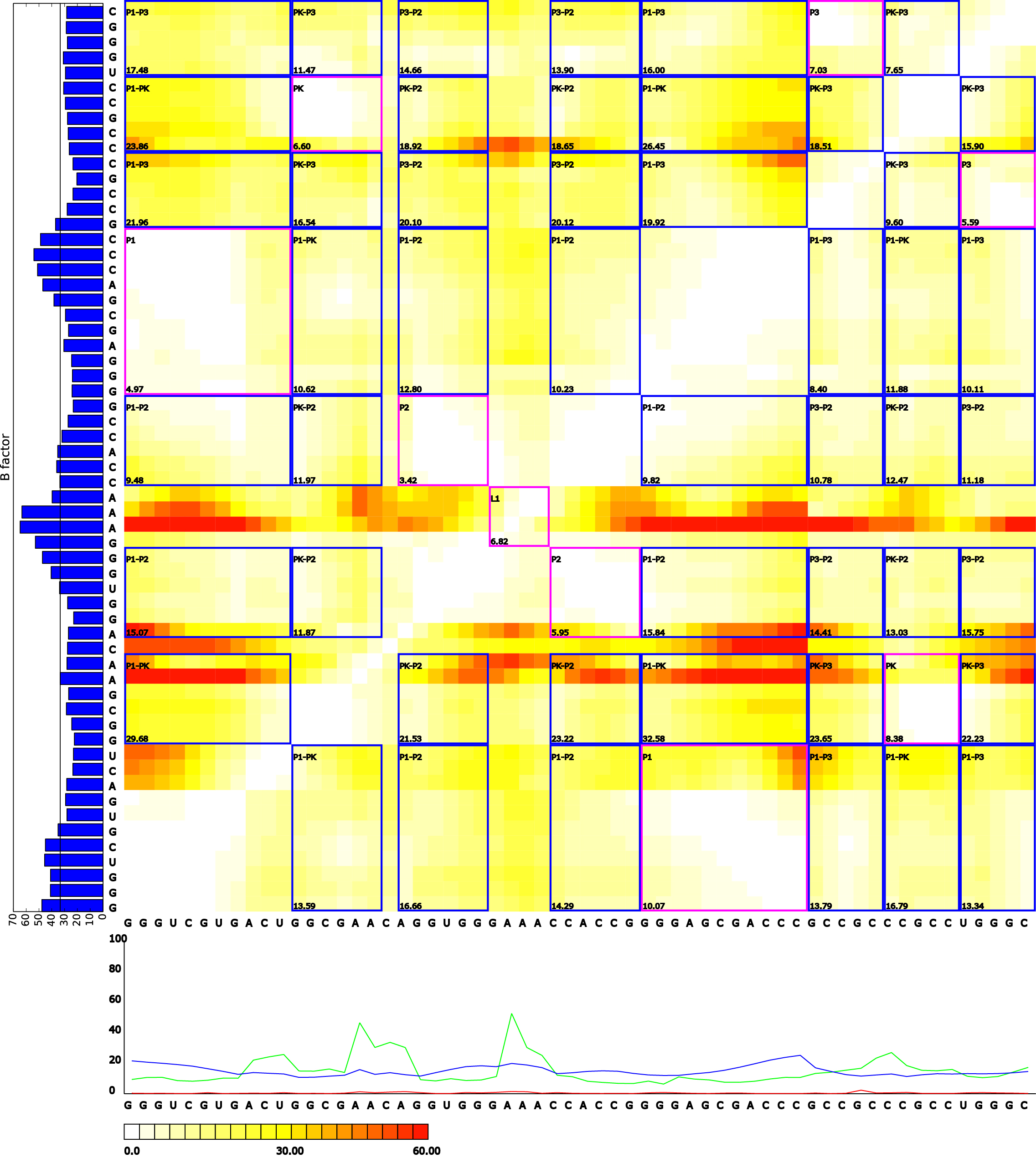

Supplement: Supplemental Material [file supp_060368.116_Supp_Fig_S9_Puzzle13-2.jpg]

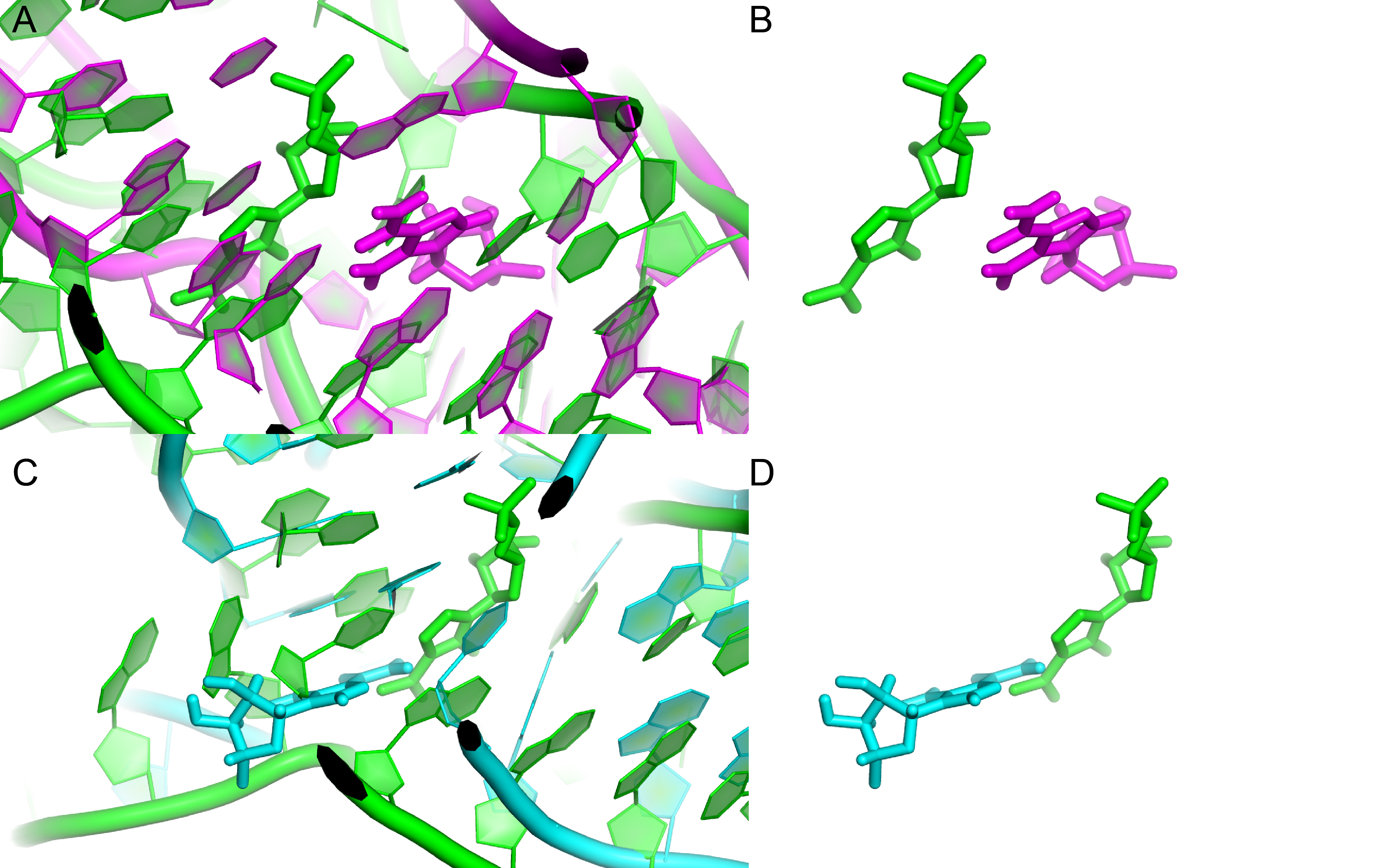

Supplement: Supplemental Material [file supp_060368.116_Supp_Fig_S10.jpg]

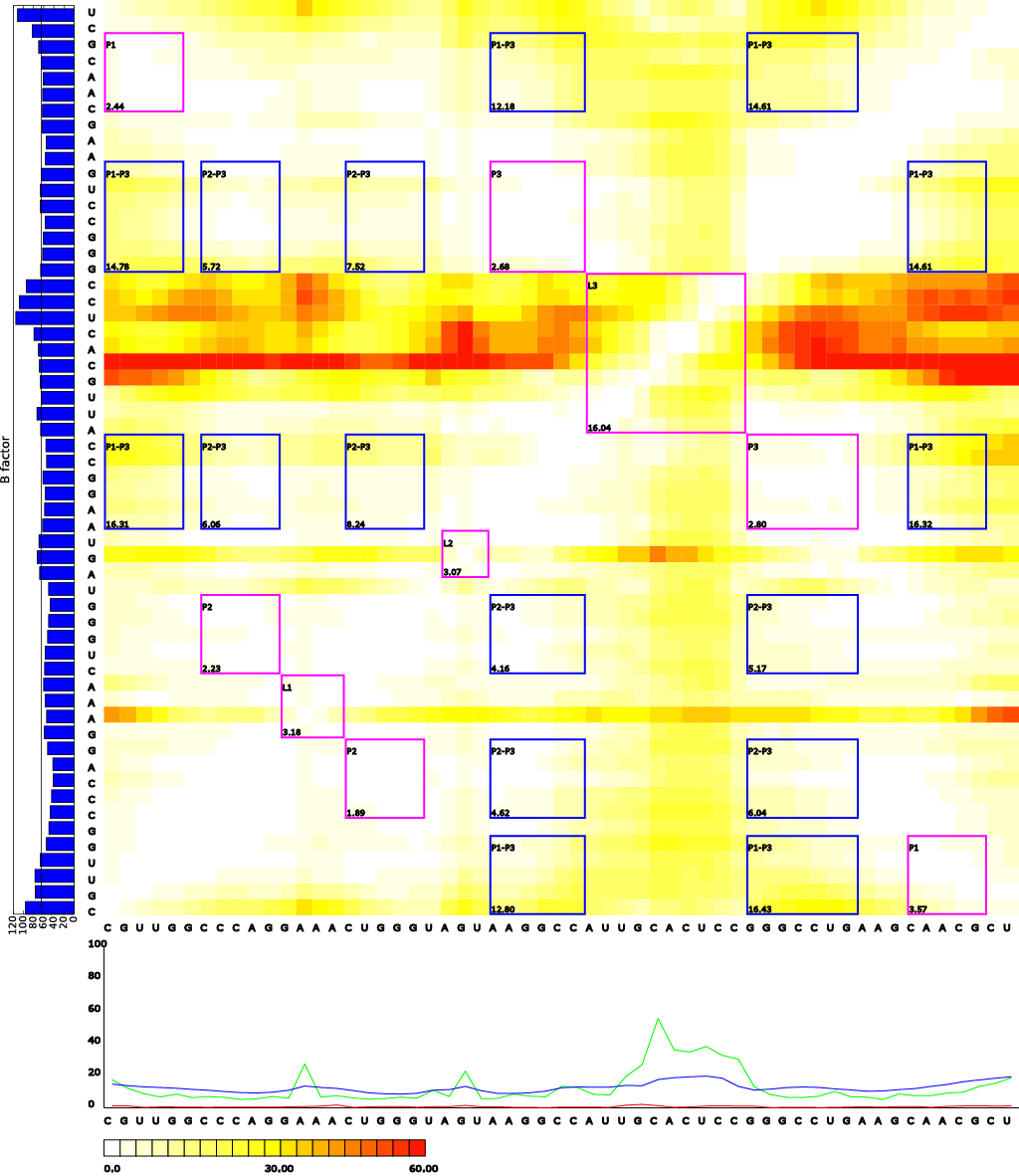

Supplement: Supplemental Material [file supp_060368.116_Supp_Fig_S11_Puzzle14-Das2.jpg]

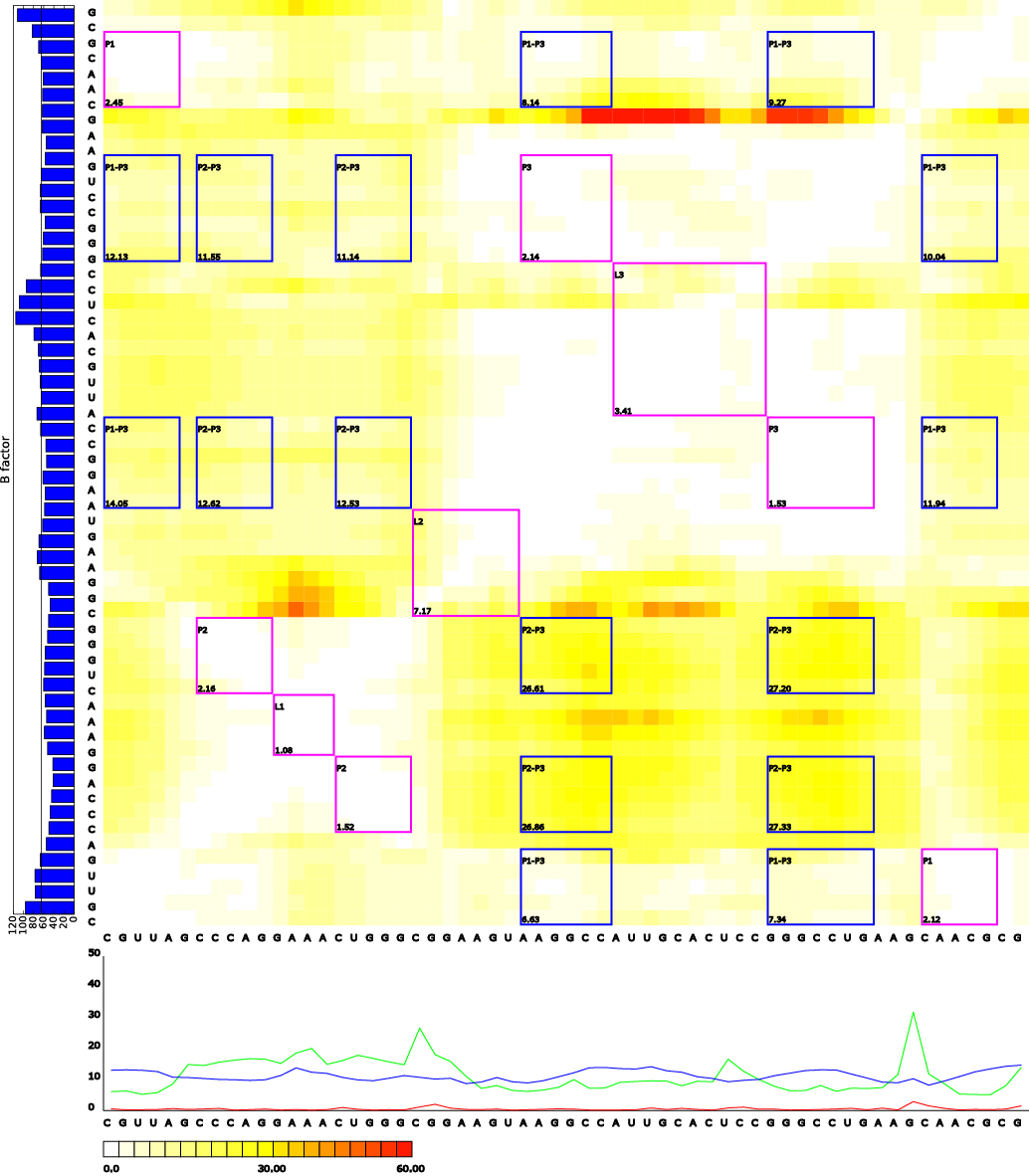

Supplement: Supplemental Material [file supp_060368.116_Supp_Fig_S12_Puzzle14-Bound_Buj2.jpg]

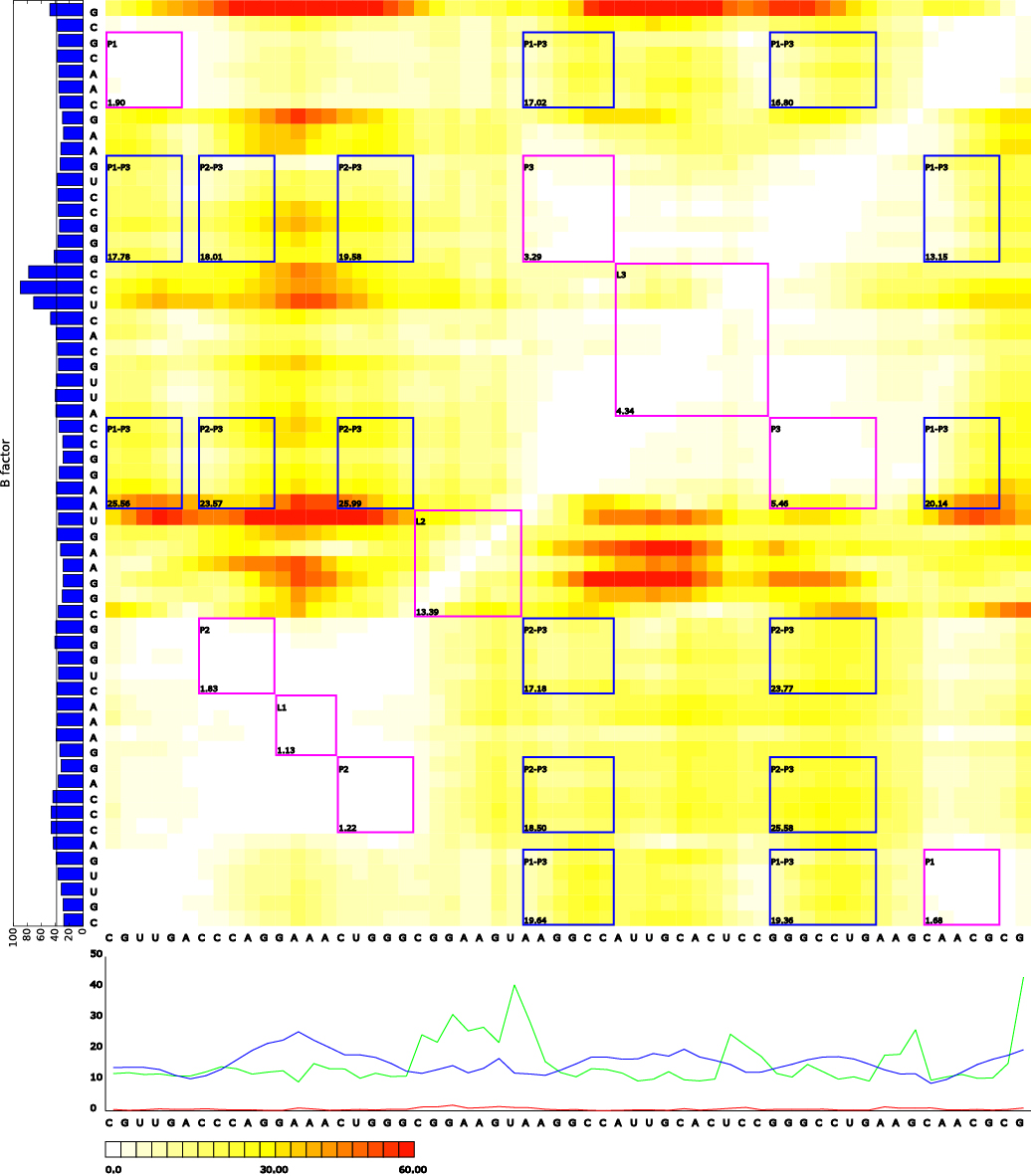

Supplement: Supplemental Material [file supp_060368.116_Supp_Fig_S13_Puzzle14-Bound_chen5.jpg]

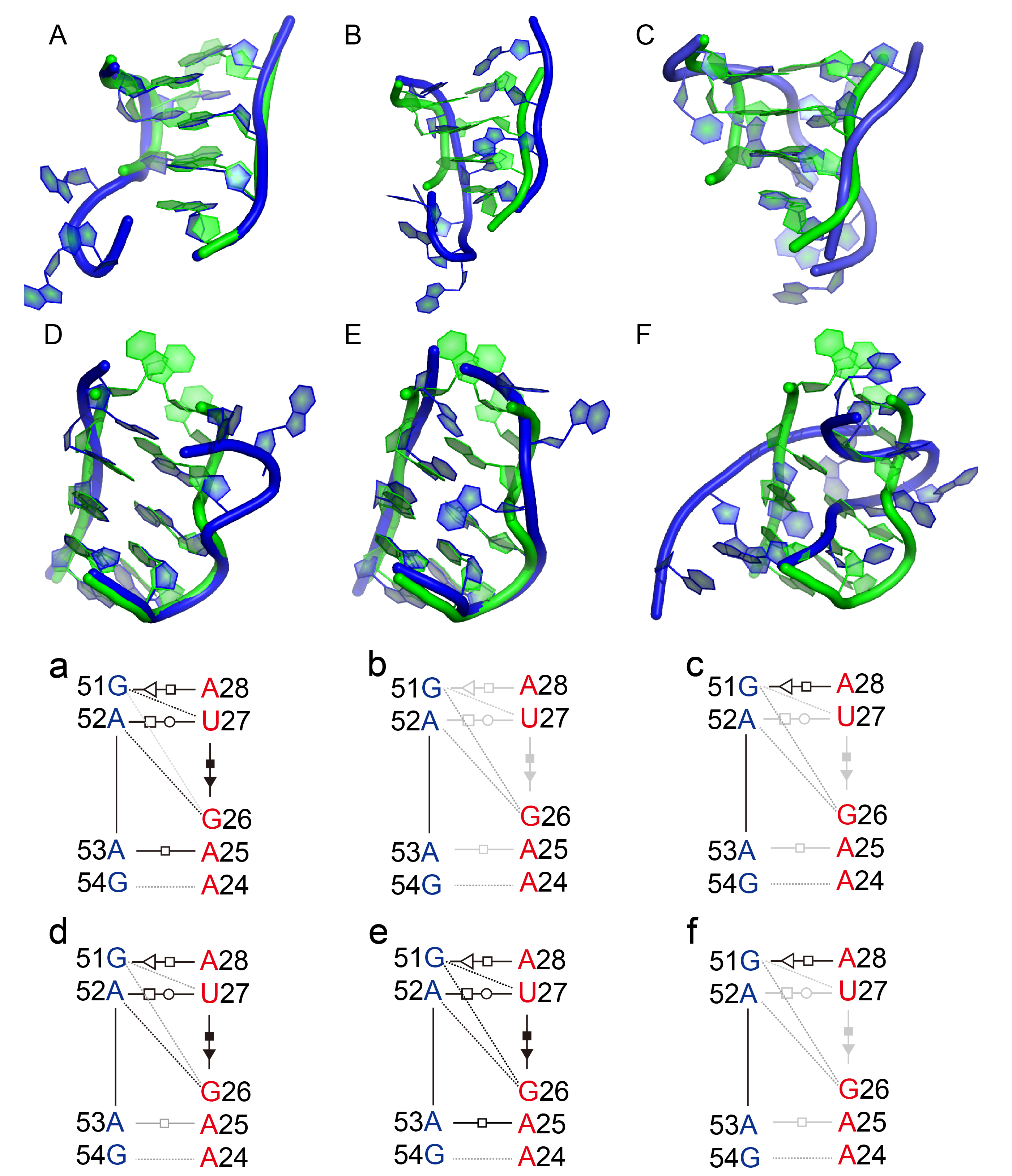

Supplement: Supplemental Material [file supp_060368.116_Supp_Fig_S14.jpg]

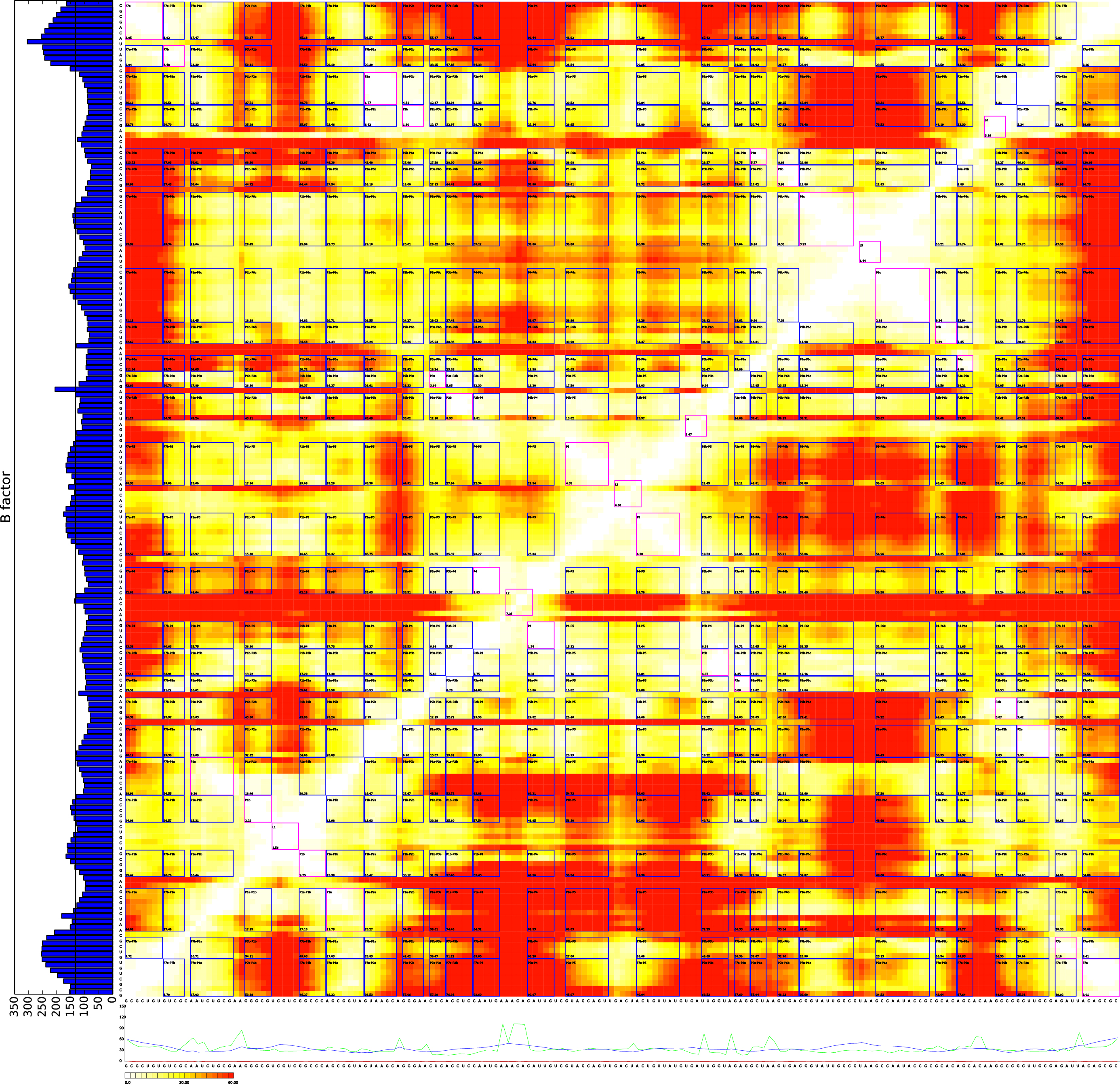

Supplement: Supplemental Material [file supp_060368.116_Supp_Fig_S15_Puzzle7-2.jpg]

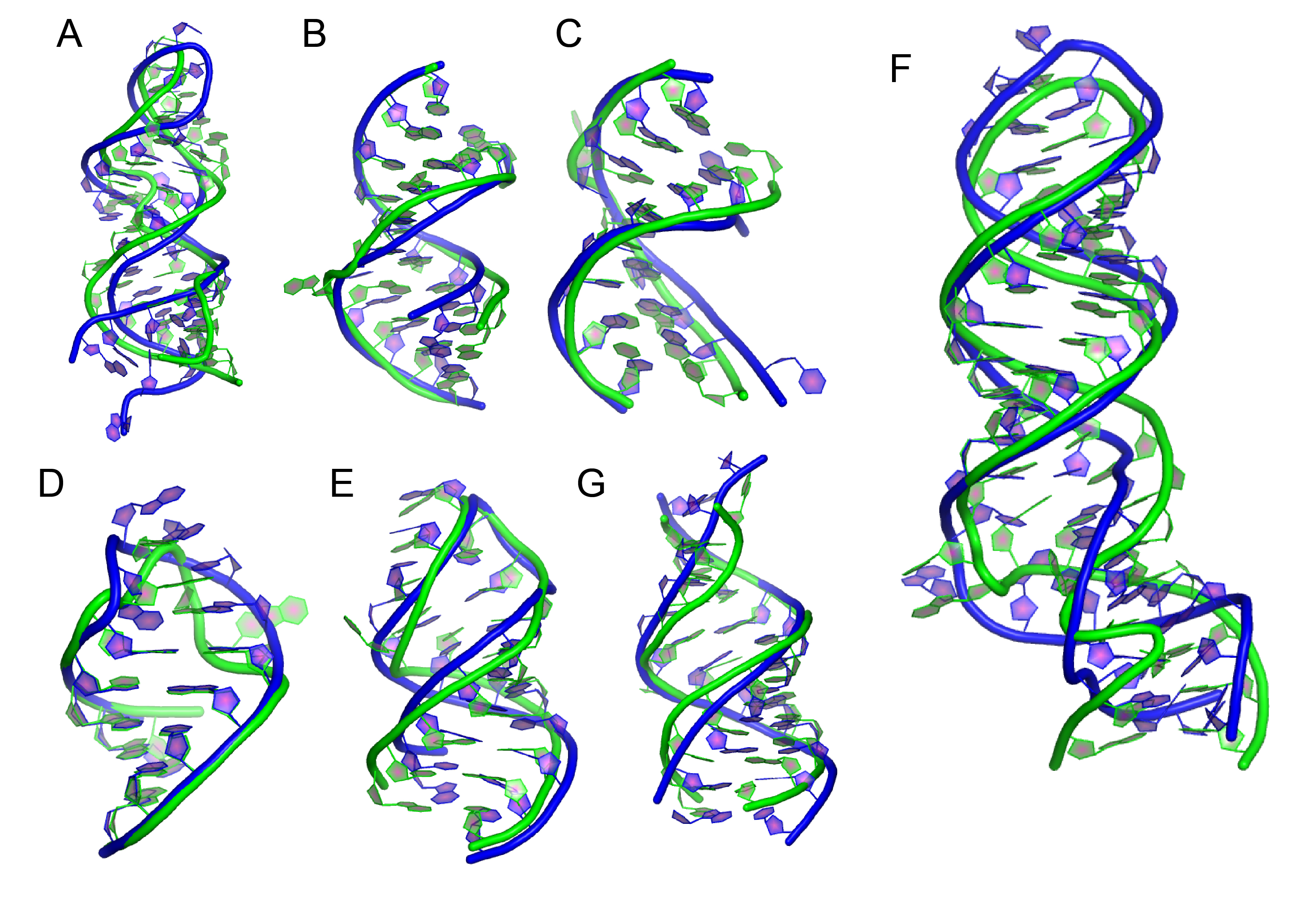

Supplement: Supplemental Material [file supp_060368.116_Supp_Fig_S16.jpg]

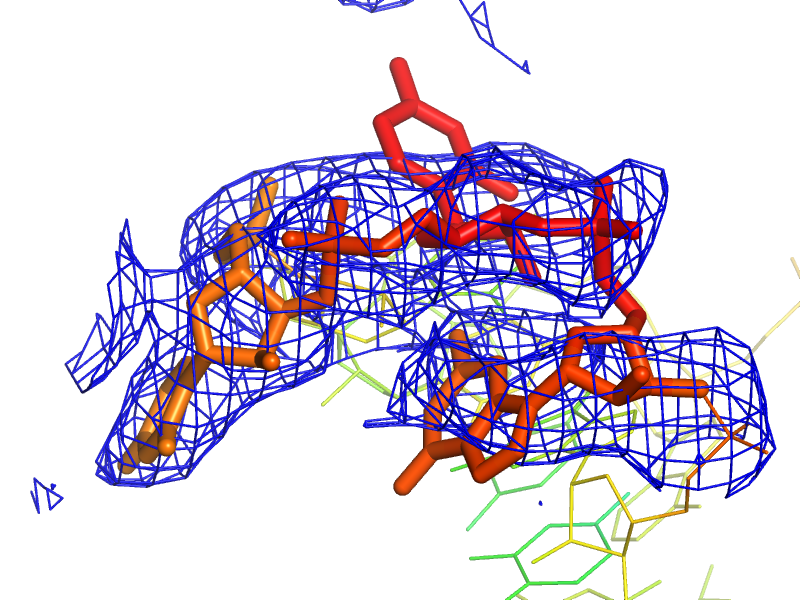

Supplement: Supplemental Material [file supp_060368.116_Supp_Fig_S17.png]

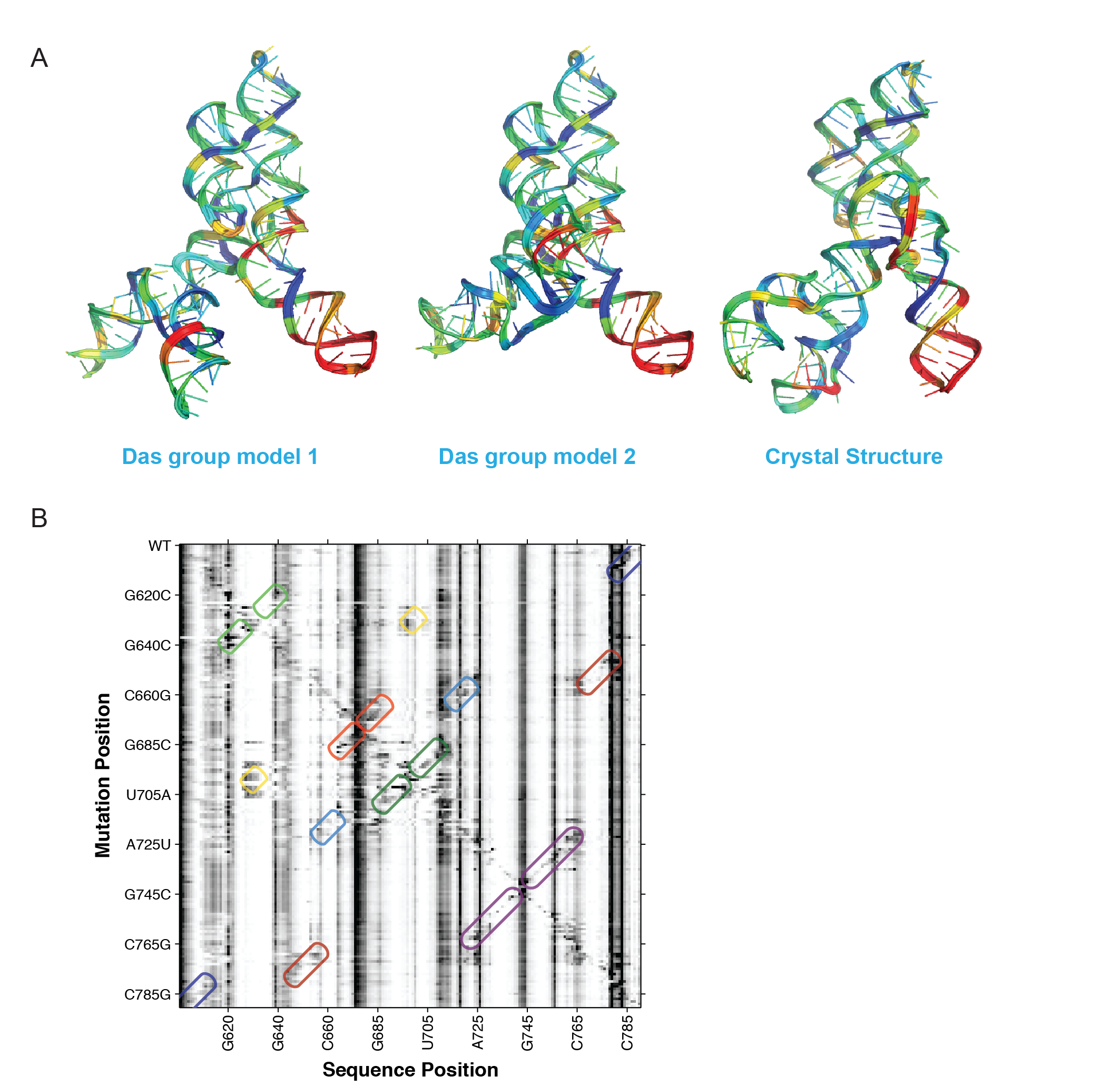

Supplement: Supplemental Material [file supp_060368.116_Supp_Fig_S18.png]

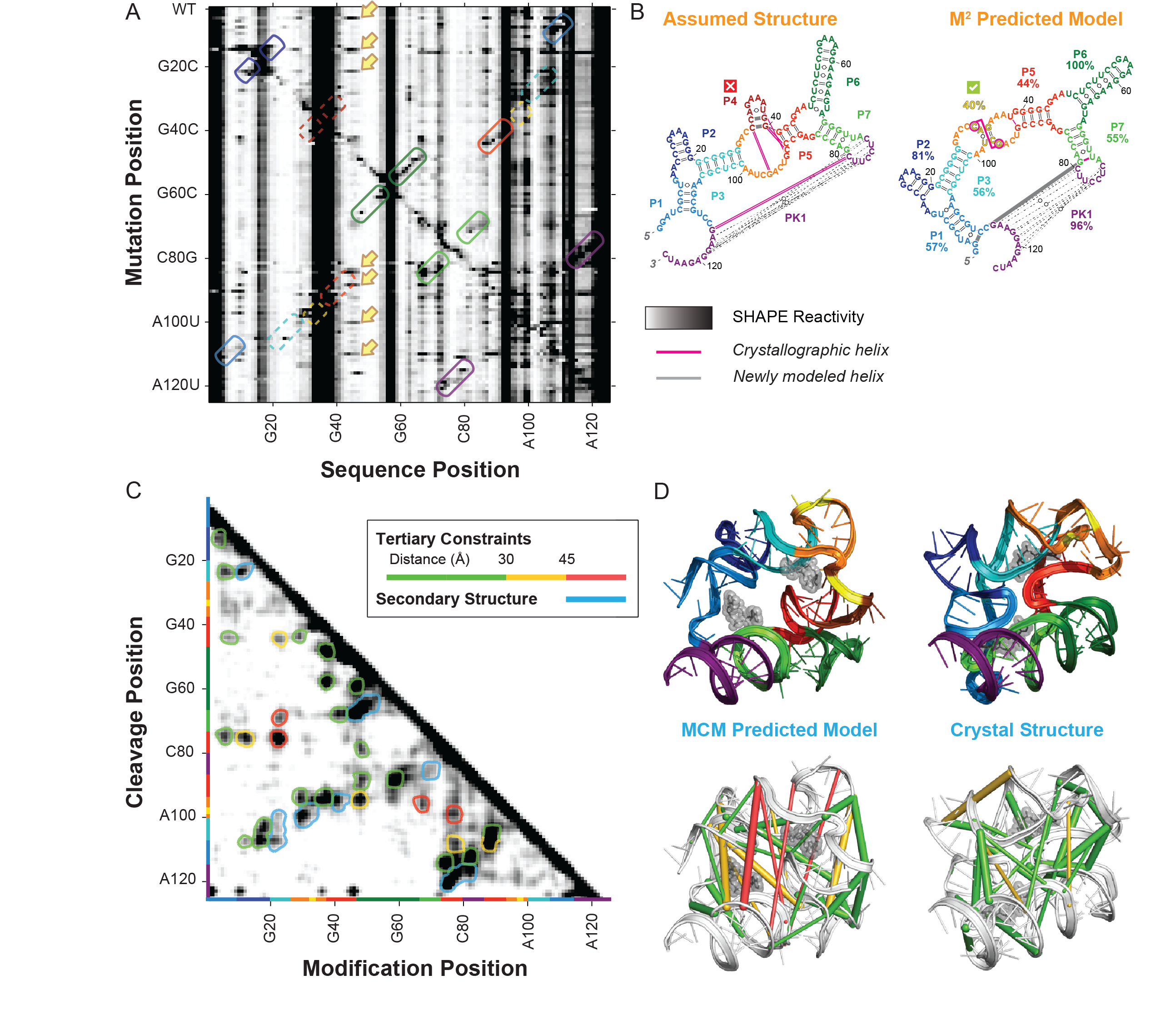

Supplement: Supplemental Material [file supp_060368.116_Supp_Fig_S19.png]

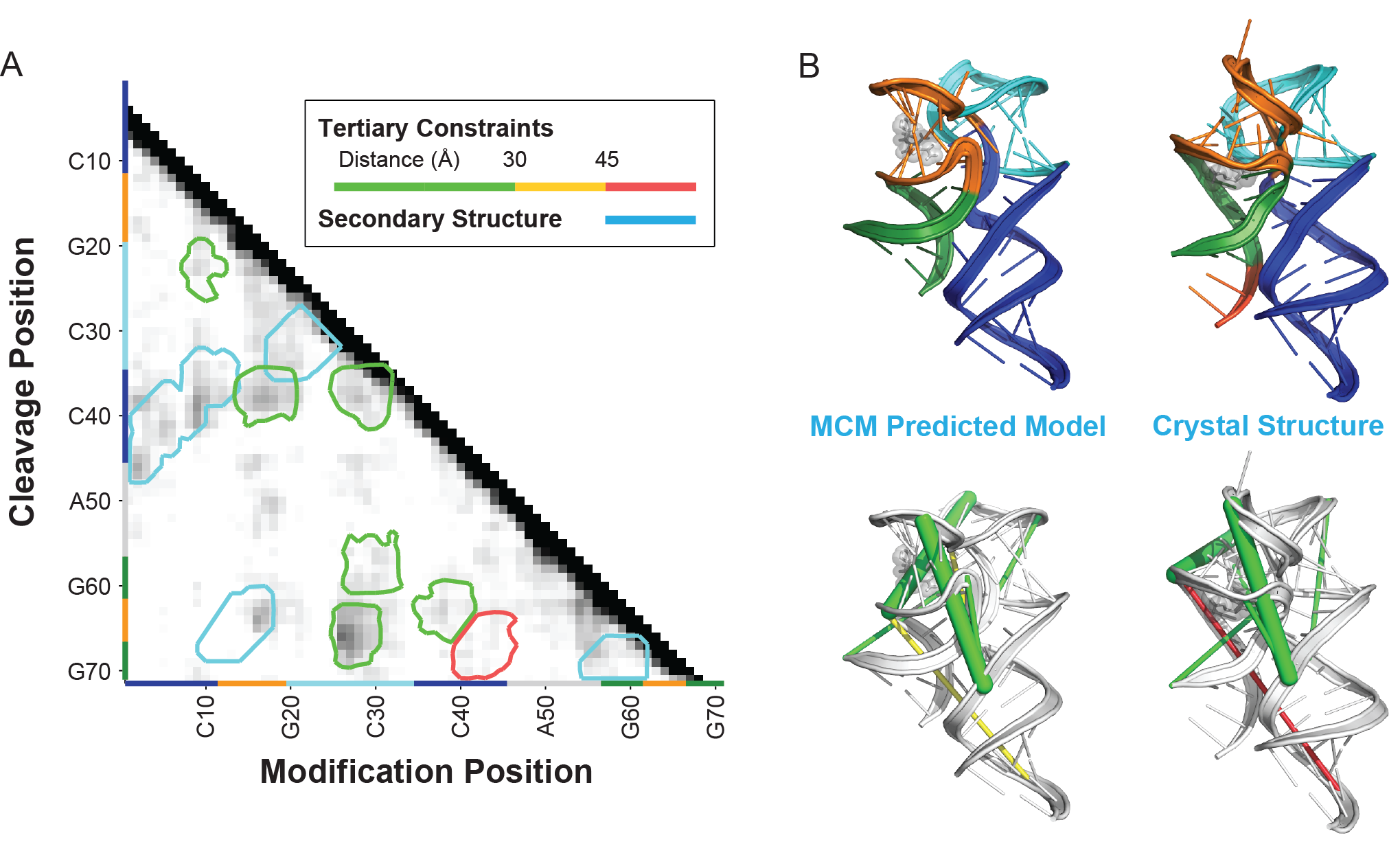

Supplement: Supplemental Material [file supp_060368.116_Supp_Fig_S20.png]
