# Supplementary material for: RNA-Puzzles Round III: 3D RNA structure prediction of five riboswitches and one ribozyme
Source: RNA. 2017 May;23(5):655–72. doi: 10.1261/rna.060368.116 (PMC5393176; doi:10.1261/rna.060368.116)
Supplement: Supplemental Material [file supp_060368.116_Supp_Figs_S21-S27.docx]

Radar diagrams allow to interpret visually multiple rankings at the same time. They facilitate discovering a dominance relation if some model is better than the other on all the criteria. In such a case, a closed contour line corresponding to the dominated model is contained inside its dominant. Radar diagrams also clearly show each selected model’s strong and weak points, as far as the related scores are considered. However, one should be aware that relative rankings, although very useful in providing a quick insight into the relationship between the models, do not show the model quality with respect to a target structure. Thus, raw data should be always examined for detailed information.

**Figures S21-S27** present radar diagrams for Puzzles 4, 7, 8, 12, 13 and 14, respectively. Every figure contains left and right diagram that reflects a relative ranking of selected models versus all models submitted in the respective puzzle. In order to draw a single diagram we ranked all models submitted in the puzzle, according to the following scores: RMSD, MCQ, INF stacking, INF nwc, INF wc, INF all. Thus, six score-related rankings were obtained for each puzzle. Next, we selected one model representing every participant’s submission and we illustrated its position in the ranking over all models. In particular, each left diagram aims to show a place of every participant’s model 1 in six rankings. Right diagram pictures places of models with the best RMSD values.

A single contour line in the radar diagram is associated with one model. It crosses six data points corresponding to six score-related rankings. Outermost data point reflects high position of the model in the respective ranking (i.e. it is achieved by model with the smallest RMSD/MCQ value or the biggest INF). Similarly, the center point corresponds to the worst score. Let us add that in the case of INF-based scores, models lacking true positives were placed at the end of the related rankings.

Every participant group is assigned a unique color (see figure legends) that facilitates its model identification on every diagram. Since not all the groups participated in all considered puzzles, not all colors appear in every figure.

Legend


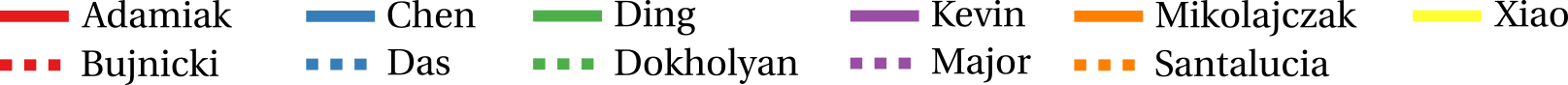


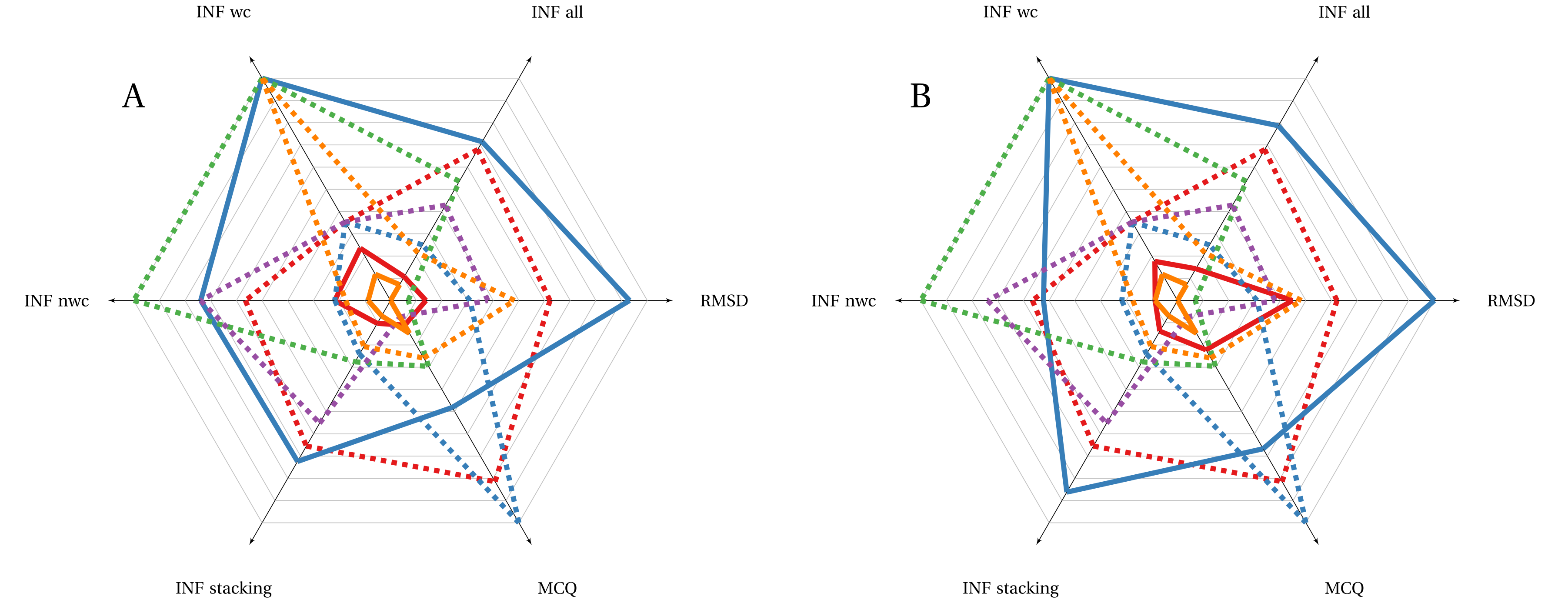


**Figure S21**: Ranking of models in Puzzle 4


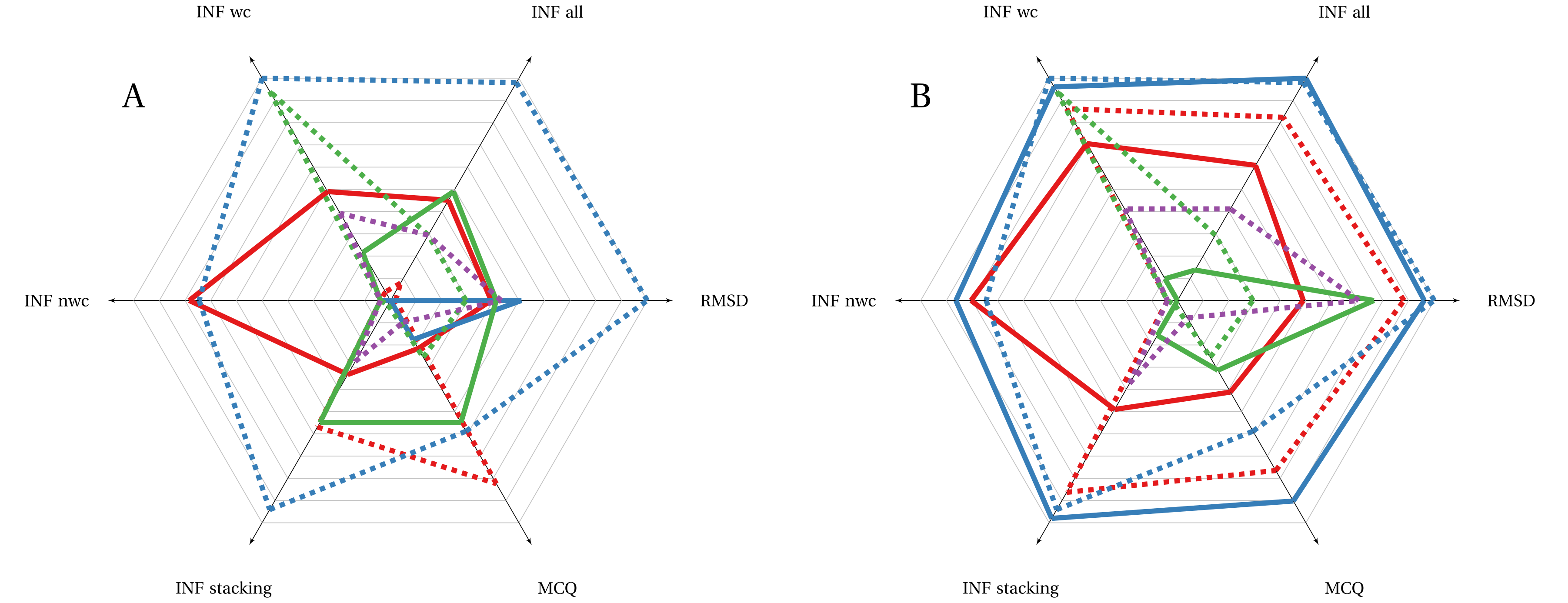


**Figure S22**: Ranking of models in Puzzle 7

**
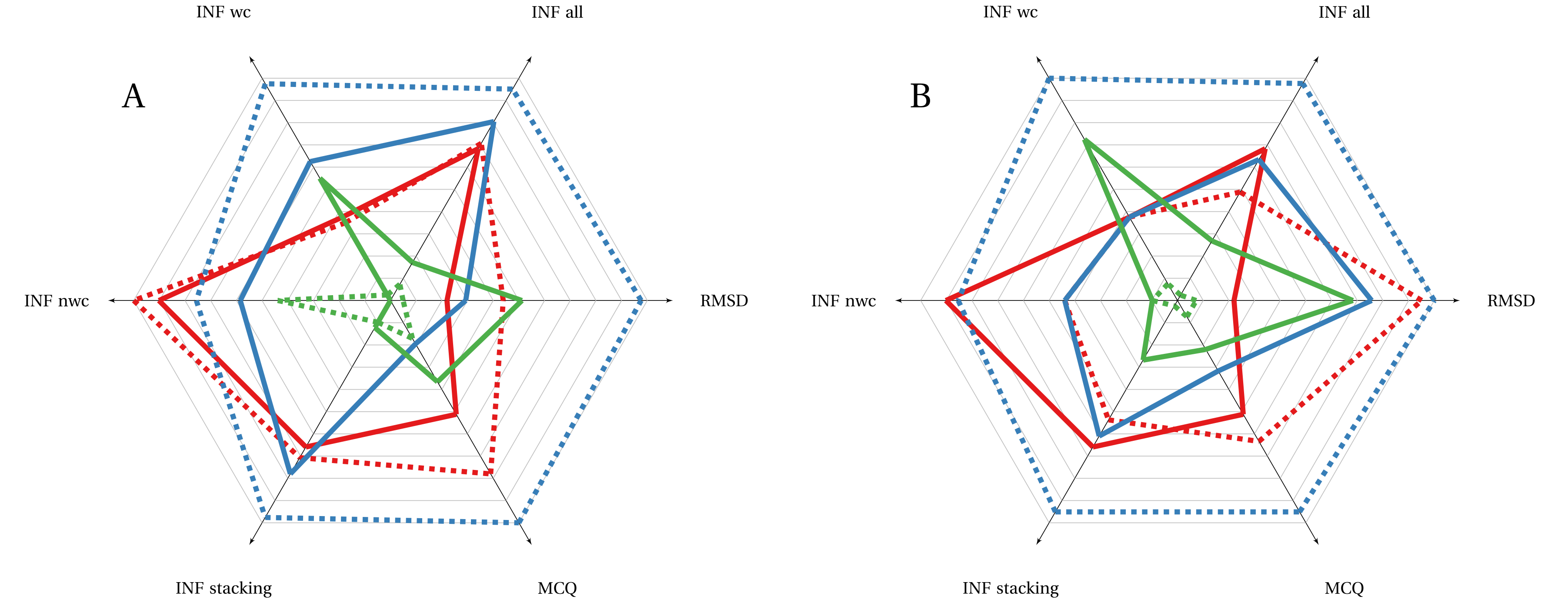
**

**Figure S23**: Ranking of models in Puzzle 8


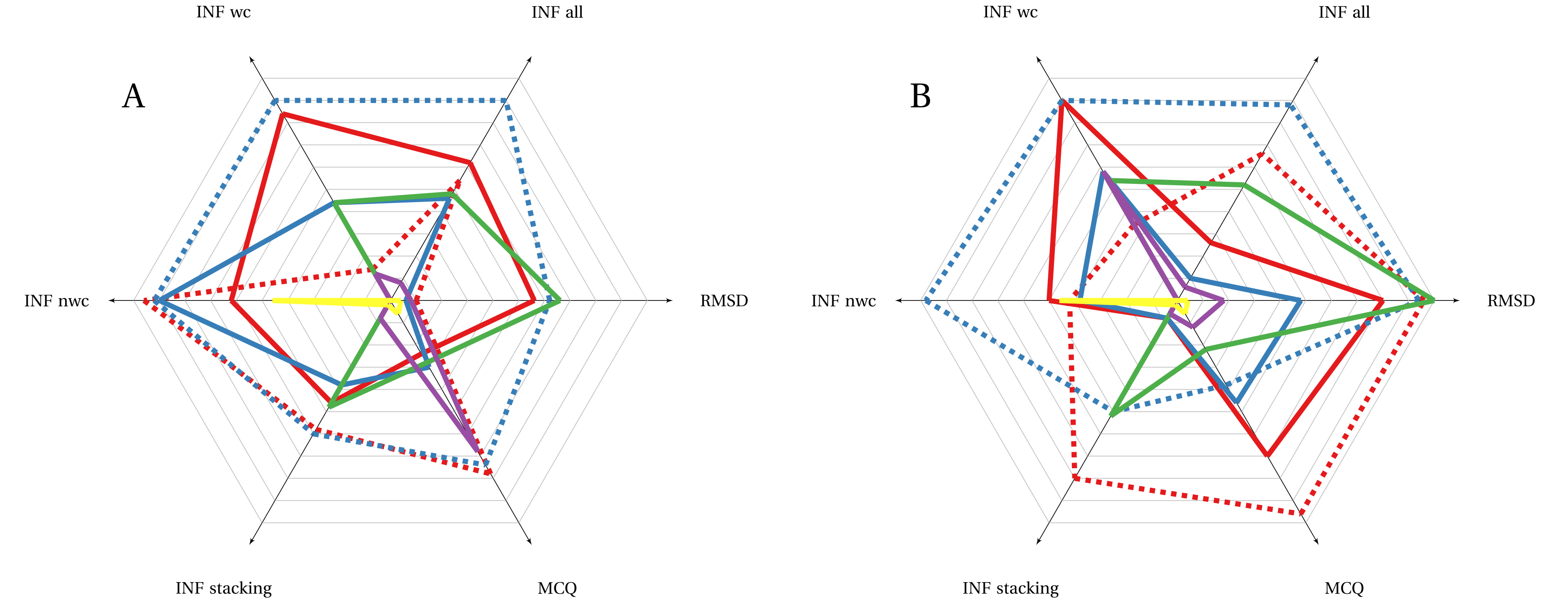


**Figure S24**: Ranking of models in Puzzle 12


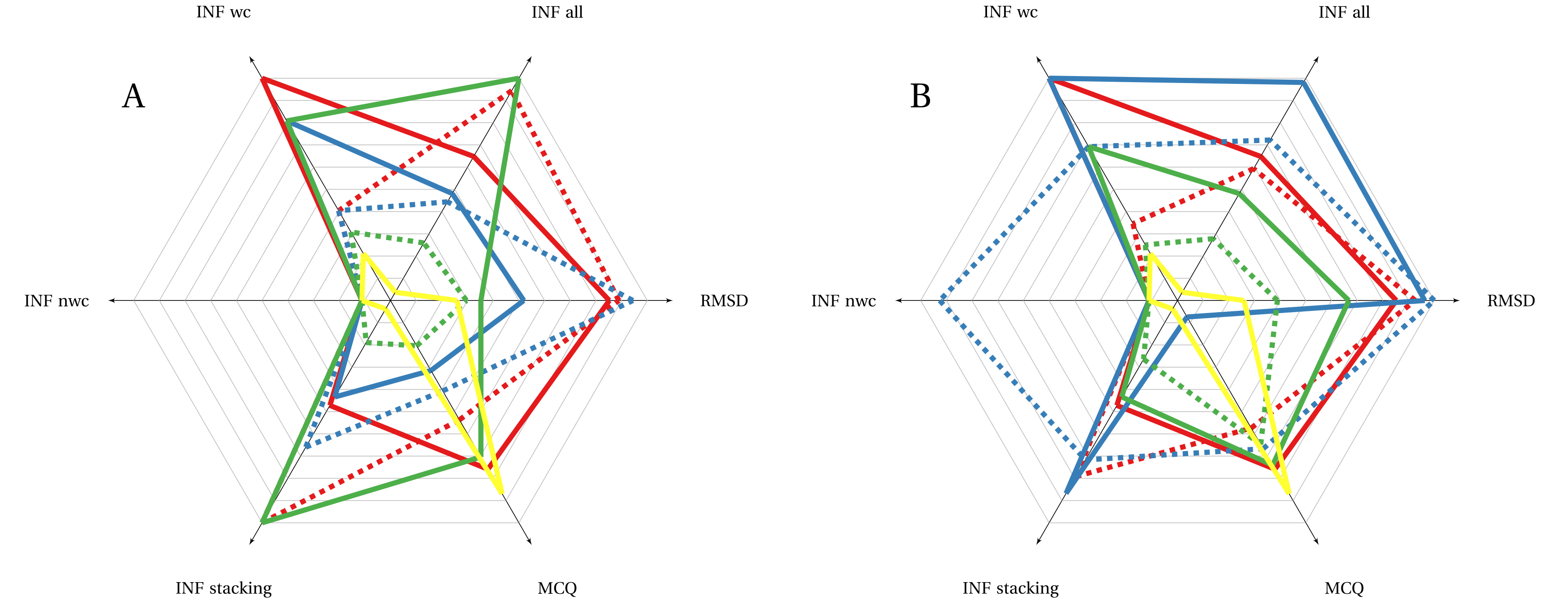


**Figure S25**: Ranking of models in Puzzle 13

Legend

**
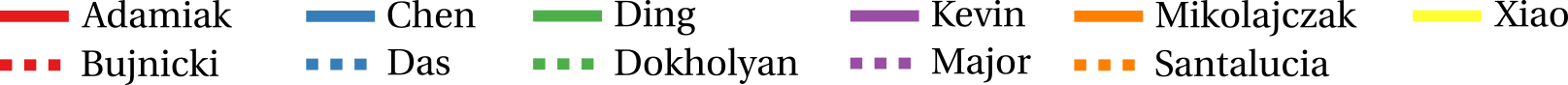
**

**Figures** S21-S25 show rankings of selected models in *Puzzles* 4, 7, 8, 12 and 13, respectively. Radar diagrams on the left (A) represent the relationship between models submitted as model 1 by each group. Diagrams on the right (B) were prepared for models with the best RMSD value for the current group.

One can observe that in majority of cases there is no model to outperform all the others on all the scores. For example, Das model 1 in *Puzzle* 7 is the best on all but one (MCQ) scores and almost the same in terms of INF nwc as Adamiak model 1 (Figure S22A). In MCQ-based ranking it is outperformed by Bujnicki model 1. In the same puzzle, Chen model 1 has quite good RMSD score, but it is totally far behind the others in the remaining ranks. On the other hand, Chen model 5 submitted for this puzzle is clearly the best on almost all scores (Figure S22B). Diagrams for *Puzzle* 13 show that neither group predicted non-canonical base pairs precisely. Thus, all plots in Figure S25A cross the INF nwc axis very close to the center (zero point).


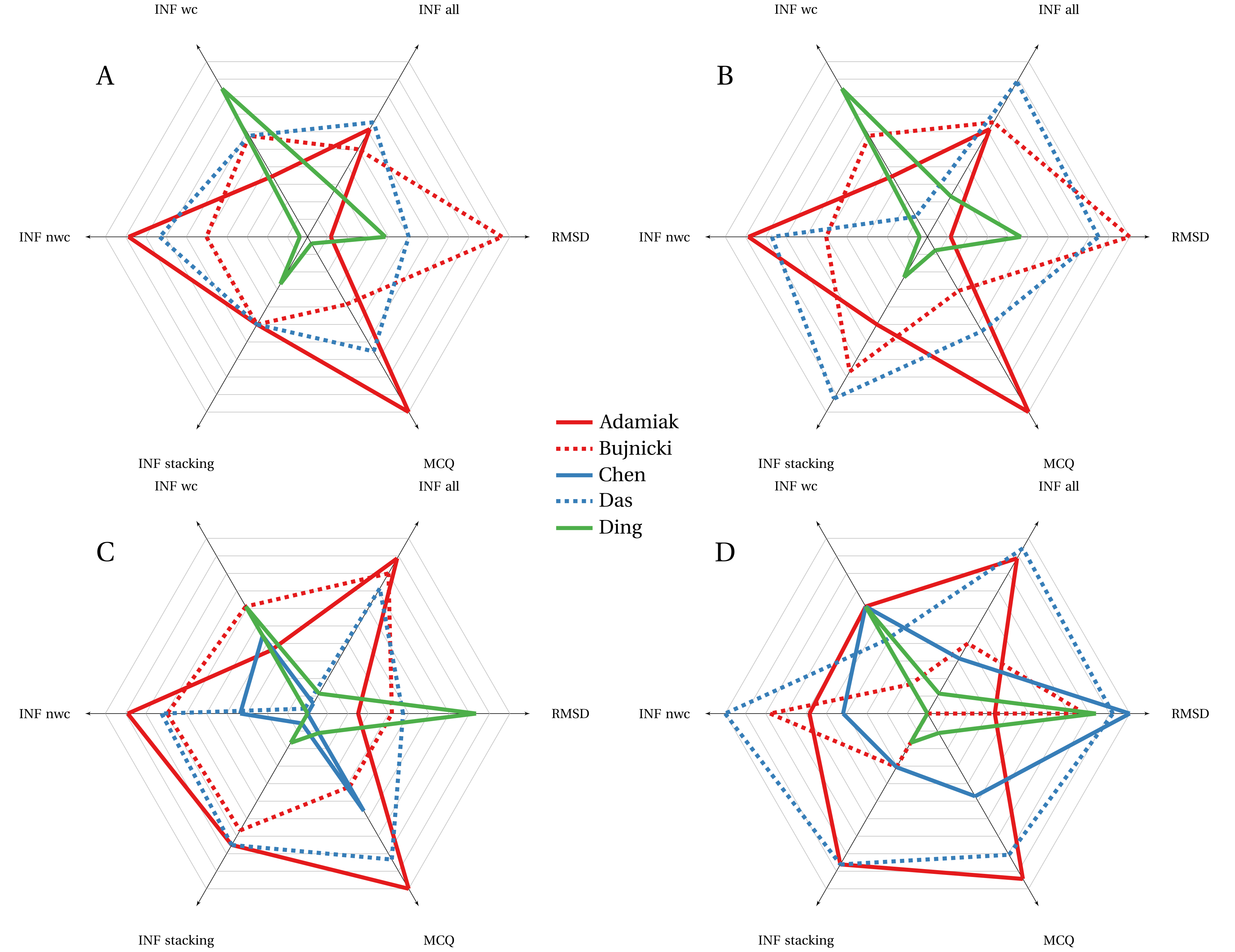


**Figure S26**: Ranking of models in Puzzle 14 (bound state)


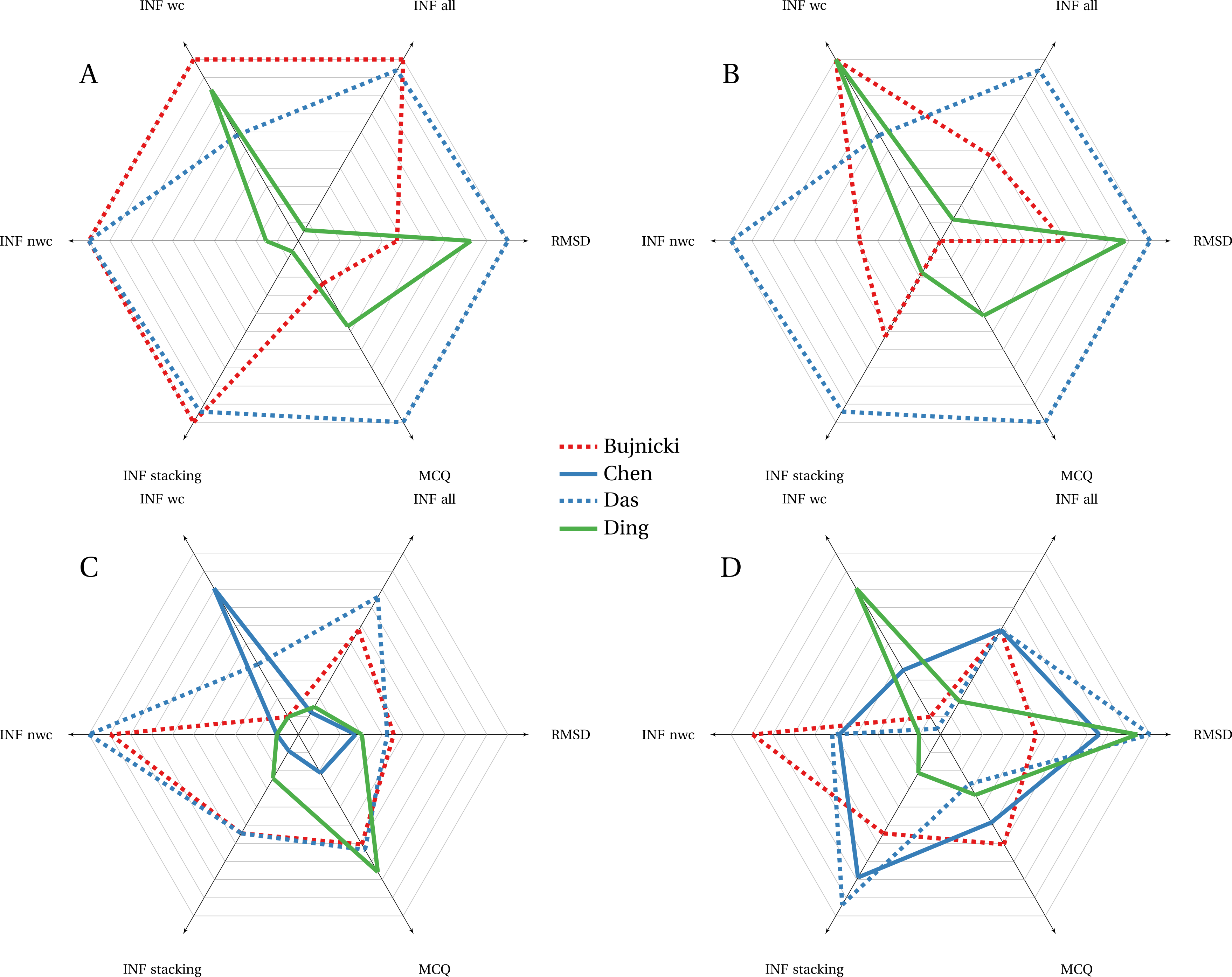


**Figure S27**: Ranking of models in Puzzle 14 (free state)

**Figures** S26-S27 present rankings for *Puzzle* 14 in bound and free state, respectively. Diagrams A and B represent the state before experimental data were available, while C and D show results of structure modelling including this data. Interestingly, in several cases, an addition of experimental restraints worsened particular model ranking.
