# Supplementary material for: RNA-Puzzles Round III: 3D RNA structure prediction of five riboswitches and one ribozyme
Source: RNA. 2017 May;23(5):655–72. doi: 10.1261/rna.060368.116 (PMC5393176; doi:10.1261/rna.060368.116)
Supplement: Supplemental Material [file supp_060368.116_Supp_Tables_S8-S9.docx]

**Table S8**. Accession IDs for chemical mapping data from RMDB

|  | 1D | 2D | MOHCA |
| --- | --- | --- | --- |
| Puzzle 7 | RNAPZ7_STD_0000 | RNAPZ7_1M7_0001  RNAPZ7_DMS_0001  RNAPZ7_NMD_0001 | RNAPZ7_MCA_0000  RNAPZ7_MCA_0001 |
| Puzzle 8 | RNAPZ8_STD_0001  RNAPZ8_HRF_0001 | RNAPZ8_1M7_0001  RNAPZ8_CMCT_0001  RNAPZ8_DMS_0001  RNAPZ8_NMD_0001 |  |
| Puzzle 12 | RNAPZ12_1M7_0000  RNAPZ12_CMC_0000  RNAPZ12_DMS_0000 | RNAPZ12_1M7_0003  RNAPZ12_DMS_0003 | RNAPZ12_MCA_0000  RNAPZ12_MCA_0001  RNAPZ12_MCA_0002  RNAPZ12_MCA_0003 |
| Puzzle 13 | RNAPZ13_STD_0000  RNAPZ13_HRF_0000 | RNAPZ13_1M7_0001  RNAPZ13_1M7_0002  RNAPZ13_1M7_0003  RNAPZ13_DMS_0001  RNAPZ13_DMS_0002  RNAPZ13_DMS_0003  RNAPZ13_NMD_0003 | RNAPZ13_MCA_0000  RNAPZ13_MCA_0001 |
| Puzzle 14 | RNAPZ14_STD_0000  RNAPZ14_STD_0001  RNAPZ14_HRF_0000  RNAPZ14_HRF_0001 | RNAPZ14_1M7_0000  RNAPZ14_1M7_0001  RNAPZ14_1M7_0002  RNAPZ14_1M7_0003 | RNAPZ12_MCA_0000  RNAPZ12_MCA_0001  RNAPZ12_MCA_0002  RNAPZ12_MCA_0003 |

**Table S9**. The RNAComposer input data for *RNA Puzzle* challenges

| *Puzzle* # | Input RNA secondary structure topology | No of introduced restraints |
| --- | --- | --- |
| *Puzzle* 4 | ((((((((....(.(((...(((...[[)))......))))(((..(((((((((((((((((((....))))))))))))))))))).)))...(]]..(((((....)))))..))))))))). | 89 |
| *Puzzle* 8 | (((((....((((....)))).((.(((((((.[[[[[.)))).)))))...(((((....)))))))))).((((....))))......]]]]]. | 510 |
| *Puzzle* 12 | (((.((((...(((....)))((((((....((....))(((.....(((((((....)))))))..(((((.[[[[[[.)))))...))).........)))))).))))))).]]]]]].... | 747 |
|  | (((.((((...(((....)))((((((....((....))((((....(((((((....)))))))..(((((.[[[[[[.)))))..)))).........)))))).))))))).]]]]]].... | 747 |
|  | (((.((((...(((....)))((((((....(......)((((....(((((((....)))))))..(((((.[[[[[..)))))..)))).........)))))).)))))))..]]]]].... | 525 |
| *Puzzle* 13 | (((((((....[[[[....(((((....))))).....)))))))...........((((..]]]]).))) | 329 |
| *Puzzle* 14 | (((((.(((((....)))))........((((((..........))))))....))))).. | 0 |
| *Puzzle* 7 | ((((((((((.....((((...(((....[[[[[)))..))))..((((((.(((..((((((..(((((.....))))).(((((((.]]]]]...))))))).....))).)))(((..((..((((((((((....))))))))))..))..))).....)))))))))..))).))))))) | 512 |
|  | ((((((((((.....((((...(((....[[[[[)))..))))..((((((.(((..((((((..(((((.....))))).(((((((.]]]]]...))))))).....))).)))(((..((..((((((((((....))))))))))..))..))).....)))))))))..))).))))))) | 512 |
|  | ((((((((((.....((((...(((....[[[[[)))..))))..((((((.(((..((((((..(((((.....))))).(((((((.]]]]]...))))))).....))).)))(((..((..((((((((((....))))))))))..))..))).....)))))))))..))).))))))) | 512 |
|  | ((((((((((.....((((...((..[[.[[[[[.))..))))..((((((.(((..((((((..(((((.....))))).((((((..]]]]].]].)))))).....))).)))(((..((..((((((((((....))))))))))..))..))).....)))))))))..))).))))))) | 602 |
|  | ((((((((((.....((((...((((...[[[.))))..))))..((((((.(((..((((((..(((((.....))))).(((((((((.]]].))))))))).....))).)))(((..((..((((((((((....))))))))))..))..))).....)))))))))..))).))))))) | 193 |
